# Adolescents’ experience of living with X-linked hypophosphataemia (XLH): a mixed-methods analysis of those who continued and discontinued burosumab treatment after end of skeletal growth

**DOI:** 10.1186/s13023-026-04244-2

**Published:** 2026-02-03

**Authors:** Vrinda Saraff, Pedro Arango-Sancho, Justine Bacchetta, Annemieke M. Boot, Christine P. Burren, Amish Chinoy, Poonam Dharmaraj, Maria Amelia Gómez Llorente, Juan David González Rodríguez, Iva Gueorguieva, Wesley Hayes, Dirk Schnabel, Héctor Ríos Duro, Elin Haf Davies, Sandra Komarzynski, Angela J. Rylands, Kerry Sandilands, Haruka Ishii, Angela Williams, Santhani Selveindran, Adele Barlassina, Annabel Bowden, Agnès Linglart

**Affiliations:** 1 Birmingham Women’s and Children’s Hospital, Birmingham, UK; 2 Sant Joan de Déu Barcelona Hospital, Barcelona, Spain; 3 Hospices Civils de Lyon, Lyon, France; 4 University Medical Center Groningen, University of Groningen, Groningen, The Netherlands; 5 University Hospitals Bristol and Weston NHS Foundation Trust, Bristol, UK; 6 Royal Manchester Children’s Hospital, Manchester, UK; 7 Alder Hey Children’s Hospital, Liverpool, UK; 8 Hospital Virgen de Las Nieves, Granada, Spain; 9 Department of Pediatric Nephrology, Santa Lucia General University Hospital, Cartagena, Spain; 10 Centre Hospitalier Universitaire de Lille, Lille, France; 11 Great Ormond Street Hospital, London, UK; 12 Center for Chronic Sick Children, Pediatric Endocrinology, Charité – University Medicine Berlin, Berlin, Germany; 13 Pediatric Nephrology, Vall d’Hebron University Hospital, Barcelona, Spain; 14 Aparito Ltd, a Wholly Owned Subsidiary of Eli Lilly &Co., Wrexham, UK; 15 Kyowa Kirin International, Marlow, UK; 16 Kyowa Kirin Co., Ltd., Tokyo, Japan; 17 Open Health Ltd, Marlow, UK; 18 Spire Outcomes Limited, London, UK; 19 Paris Saclay University, AP-HP, INSERM Bicêtre Paris Saclay Hospital, Le Kremlin Bicêtre, Paris, France

**Keywords:** X-linked hypophosphataemia, Rare disease, Patient-reported outcomes, Paediatrics, Puberty, Health-related quality of life (HRQL), Qualitative research, Fibroblast growth factor 23 (FGF23), Phosphate wasting

## Abstract

**Background:**

X-linked hypophosphataemia (XLH) is a rare, genetic, phosphate-wasting disorder caused by excess fibroblast growth factor 23 (FGF23). Children experience skeletal abnormalities, pain and impaired health-related quality of life (HRQL). The FGF23 inhibitor burosumab improved growth, decreased rickets severity and improved symptoms and HRQL in paediatric phase 3 trials. Using a mixed-methods approach, the MyXLH study aims to describe the lived experience of adolescents with XLH and to compare the experiences of adolescents who did and did not continue burosumab for the 26 weeks immediately after end of skeletal growth (EoSG), based on patient-reported daily activity, symptoms and HRQL, enriched with telephone interviews.

**Results:**

Twenty-five adolescents were enrolled (16 girls, 9 boys) at centres in France, Germany, the Netherlands, Spain and the UK. EoSG (confirmed mostly by growth velocity and/or imaging) occurred at a mean (SD) age of 15.7 (1.3) years in girls and 17.2 (0.7) years in boys. Mean (SD) time on burosumab before EoSG was 4.3 (1.9) and 4.9 (2.6) years in those who continued and discontinued burosumab (*n* = 16 and 9), respectively. In adolescents who continued burosumab, serum phosphate levels remained stable after EoSG. Scores for Worst Pain, Worst Stiffness and Worst Fatigue were low and changed little, and physical activity (daily step count) was maintained. Mean EuroQol 5-dimension, 3-level youth (EQ-5D-Y-3 L) utility scores were 0.86 (0.24) before EoSG (*n* = 15) and 0.77 (0.30) after (*n* = 5). In interviews, these adolescents reported participating in school, physical and leisure activities; improvements in symptoms were linked to improved emotion. In adolescents who stopped burosumab at EoSG, phosphate levels decreased to below normal, scores for Worst Pain, Stiffness and Fatigue increased slightly (worse symptoms) but step count was broadly maintained. The mean (SD) EQ-5D-Y-3 L utility score decreased from 0.94 (0.10) before EoSG (*n* = 5) to 0.84 (0.15) (*n* = 3) after. Some adolescents reported worsening or newly emergent symptoms and reduced participation in school/work, physical and social activities.

**Conclusion:**

Some adolescents experienced detrimental effects on serum phosphate and functional XLH symptoms after stopping burosumab at EoSG; continuation of burosumab beyond EoSG may therefore be warranted to maintain symptom control.

**Supplementary information:**

The online version contains supplementary material available at 10.1186/s13023-026-04244-2.

## Introduction

X-linked hypophosphataemia (XLH) is a rare, genetic, phosphate-wasting disorder caused by excess circulating levels of fibroblast growth factor 23 (FGF23) as a result of inactivating mutations in the *PHEX* (phosphate-regulating endopeptidase homologue, X-linked) gene. Excess FGF23 activity leads to renal phosphate wasting and impaired production of 1,25-dihydroxy vitamin D, which compromises intestinal phosphate absorption [1]. Persistent hypophosphataemia and dysregulated bone metabolism compromise skeletal development. Clinical manifestations of XLH, such as genu varum or genu valgus, typically appear in childhood when children start to weight-bear [2]. Walking may be delayed, gait abnormalities may be present, and children may require orthopaedic surgery [3]. Growth may be delayed or disproportionate, leading to short stature [1], and children can experience skeletal pain [1, 4], muscle pain and weakness [5, 6]. Dental mineralisation is also often impaired [7]. Bone deformities that develop during childhood become irreversible when bone growth stops, and may continue to cause pain and physical dysfunction [8], with the potential for the accumulation of further skeletal morbidities and disease burden if hypophosphataemia continues into adulthood [9, 10]. Children may have difficulty attending school and taking part in usual childhood activities [11], and the burden of XLH symptoms has a marked impact on health-related quality of life (HRQL) in adults and children, compromising psychological, emotional and physical wellbeing [12–15].

For many years, standard treatment for XLH has been oral phosphate supplementation alone or in combination with active vitamin D analogues. However, this treatment does not address the underlying causes of XLH and is rarely satisfactory in the treatment of severe disease [3]. Adherence is often suboptimal due to the inconvenience of multiple daily dosing and gastrointestinal side-effects [2, 3], and patients are at risk of hyperparathyroidism, hypercalcaemia, hypercalciuria and sequelae [9].

Burosumab is a fully human monoclonal antibody that binds to and inhibits excess circulating FGF23 activity, restoring intestinal absorption and renal reabsorption of phosphate [16]. A phase 3 trial in children aged 1–12 years demonstrated that 64 weeks’ treatment with burosumab improved growth, markers of bone biochemistry and radiological rickets severity compared with oral phosphate supplementation/active vitamin D [17] and also improved patient-reported pain interference, fatigue and HRQL [18].

Burosumab is approved by the European Medicines Agency for the treatment of XLH in children and adolescents (aged 1–17 years) and in adults. However, reimbursement/local funding varies by country and in some countries doesn’t extend to adults, requiring patients to stop treatment at the end of skeletal growth (EoSG). In other countries burosumab is available for children with growing bone and for adults, but adolescents stop treatment, at least temporarily, at EoSG.

Consensus guidelines recommend that burosumab is started as early as possible (from 1 year of age) [19, 20]. As the skeleton continues to mature and acquire bone mass beyond growth plate closure, continued treatment through adolescence to maintain adequate serum phosphate is likely to be beneficial for long-term bone health and may halt disease progression [21]. However, evidence to support the use of burosumab beyond EoSG is limited to two case series, both of which indicate that stopping treatment at EoSG is detrimental in terms of serum phosphate, physical symptoms and HRQL [22, 23]. Evidence from a small group of adults whose burosumab treatment was interrupted indicates that continued treatment is needed to maintain normal phosphate levels [24].

MyXLH is a European, observational, prospective, mixed-methods study that aimed to describe the lived experience of XLH for adolescents being treated with burosumab at EoSG [25–27]. Data collected before EoSG have already been reported [28]. The objective of the current study was to describe the experience of adolescents with XLH who did and did not continue burosumab for the 26 weeks immediately after EoSG.

## Methods

The study is reported according to the requirements of STROBE (Strengthening the Reporting of Observational Studies in Epidemiology) [29] and COREQ (Consolidated Criteria for Reporting Qualitative Research) [30] guidelines.

### Study design

MyXLH is an observational, prospective, European, multicentre, mixed-methods study; the design was informed by a structured patient engagement exercise [27]. Longitudinal quantitative and qualitative data were collected separately over the same period, analysed separately and then merged and compared for interpretation. As the study design has been reported previously [28], only the key points are summarised here.

Patients were recruited from 14 specialist paediatric centres in the UK, France, the Netherlands, Germany and Spain. Eligible patients were aged 12–17 years at enrolment, had a confirmed diagnosis of XLH (documented diagnosis of XLH and *PHEX* mutation in medical records, and evidence of at least one of hypophosphataemia and/or impaired phosphate reabsorption due to elevated FGF23), had been treated with burosumab as part of routine clinical care at a specialist paediatric centre for at least the past 12 months before study enrolment and had open growth plates at enrolment but were expected to reach EoSG within the next 26 weeks (based on the treating clinician’s judgement).

Treating clinicians predicted the date of EoSG based on routine monitoring of individual patients’ growth trajectory, in order to schedule pre-EoSG activities. They subsequently confirmed the date of EoSG according to local clinical practice, and the method used was recorded.

### Data collection

**Fig. 1:**
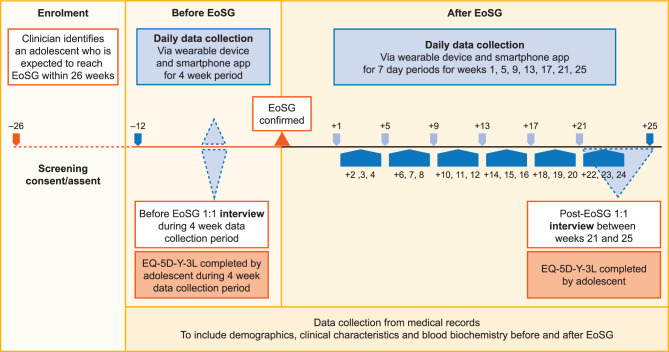
Schedule for data collection during the MyXLH study. After EoSG, some adolescents continued burosumab and some discontinued; data collection was the same for both groups. EoSG, end of skeletal growth; EQ-5D-Y-3 L, EuroQol five-dimension youth health survey

For the post-EoSG period, data were collected for 26 weeks, starting from the clinician-confirmed date of EoSG. The parameters and schedule for data collection for all adolescents are described below and summarised in Fig. 1.

### Medical records

Information relating to demographics, clinical characteristics, XLH-related treatments and biochemistry markers (phosphate, alkaline phosphatase [ALP], parathyroid hormone [PTH]) were collected from medical records (paper or electronic). Categorisation of biochemistry markers as normal or abnormal was based on local laboratory reference ranges.

### Wearable device

Adolescents were provided with a commercial wearable wrist device (Garmin Vivosmart 4) at enrolment and received training on its use. They were asked to wear the device every day for the first week of each month (weeks 1, 5, 9, 13, 17, 21 and 25) to measure the duration and intensity of physical activity (heart rate and motion intensity, including steps).

### Smartphone app

A study-specific smartphone app was developed to collect the following data:

- A diary of a typical week’s schedule of social and leisure activities during term time and holiday time
- Scores for Worst Pain, Worst Stiffness and Worst Fatigue in the last 24 hours on a scale of 0–10 (where 0 is no pain/stiffness/fatigue and 10 the worst possible); this was done daily for the first week of every month (weeks 1, 5, 9, 13, 17, 21, and 25) and once a week (at the end of the week) in between
- Pain locations (using an anatomical map) and use of analgesia
- Questions about social and leisure activities, healthcare resource utilisation (number and type of XLH-related healthcare appointments, numbers of planned and emergency appointments) and time missed from work or education (missed half or full days) during that week because of XLH
- HRQL, measured at week 25 using the EuroQol 5-dimension, 3-level youth health survey (EQ-5D-Y-3 L) [31].

Automated push reminders prompted adolescents to complete app questions. Reminders were also sent if an adolescent did not respond to survey questions on three consecutive days, together with an invitation for a telephone call with the study team.

### One-to-one semi-structured interviews

One-to-one telephone interviews were conducted with adolescents between weeks 21 and 25 using a semi-structured interview guide that aimed to explore the severity, interference, behaviours and impact on emotional wellbeing of the adolescent’s pain, stiffness and fatigue due to XLH. Adolescents’ emotional wellbeing (experiences of sadness, self-confidence, self-esteem, frustration), sleep quality, treatment, support, future aspirations and coping strategies were also discussed [27]. The interview also examined change over time and the impact of change on the social and emotional effects of experiences, response shift (adjusting perception of symptoms over time), adaptation and resilience. Adolescents were provided with their answers from a similar interview prior to EoSG to inform their reflections, in keeping with the methodology of longitudinal qualitative research [32]. This descriptive approach focuses on participants’ experiences and perspectives, rather than theory generation, and enables the researcher to stay close to participants’ viewpoints on a particular issue or phenomenon, providing a rich description of the experience [33–35].

All interviews were conducted in the local language, by experienced facilitators. Interviews were audio-recorded and audio files were translated into English using forward and backward translation and all identifiable data removed. All transcripts were uploaded to NVivo 12 (QSR International, 2018) for data management, coding and analysis.

### Safety

Safety data were collected, reported and analysed in accordance with the European Medicines Agency guidelines on good pharmacovigilance practices [36].

### Ethics statements and data protection

The study complied with all applicable laws, regulations and guidance relating to adolescent protection, including privacy, and was consistent with the ethical principles of the Declaration of Helsinki and the requirements of the European Union General Data Protection Regulation (GDPR). Ethics approval was obtained from relevant authorities in each country.

Adolescents and their carers received comprehensive information about the study and training at the start of the study by a member of the clinical team, had access to a library of study-relevant information, frequently asked questions and links to local support groups and communities via the app, and contact details for the clinical and research teams. Adolescents provided either written informed consent or assent alongside carer consent to take part in different aspects of the study, depending on age and local requirements.

All data were collected in pseudonymised form and no identifiable information was collected or removed from study centres, in order to maintain confidentiality. Only pseudonymised data were processed.

Adolescents were offered £10 (or euro equivalent) per month to cover study-related data usage and were loaned a Bluetooth-enabled smart phone for the duration of the study, if required. They were allowed to keep the wearable device after study completion. All adolescents also received a gift voucher worth £20 (or euro equivalent) in remuneration for taking part in the interview.

### Data analysis and reporting

The quantitative and qualitative data were analysed using established mixed-methods analysis [22, 37–40]. Results are reported at the group level for the adolescents who continued burosumab after EoSG. For the adolescents who discontinued burosumab, results are presented at the group level and descriptively for each individual following previous reporting formats for small heterogeneous samples [41, 42].

### Quantitative data

Continuous data are presented as mean and standard deviation (SD); categorical data are presented as numbers of patients providing data at the relevant time point (n), frequency counts and percentages. There was no statistical hypothesis testing. Analyses were performed using R, version 4.2.2.

Missing data were not imputed. At least 2 days’ data were required for calculation of means for symptoms and data from the wearable device. For the wearable device, daytime was defined as 05:00–23:00 and valid days as at least 7 hours’ daytime data.

EQ-5D-Y-3 L values were calculated according to the instrument guidelines [31]. Health state utility values were determined using the Spanish value set [43] and the EQ-5D package in R [44]. The EQ-5D-Y-3 L comprises five domain scores (mobility, looking after myself, usual activities, pain/discomfort, worried/sad/unhappy) with a reference period of ‘today’. Responses are used to determine a utility score in which a score of 1 indicates full health and a score of 0 (or less) is a state equivalent to being dead or worse than dead. Current health status is scored on a 0–100 visual analogue scale (the EQ-VAS) with a reference period of ‘today’.

### Qualitative data

Data were analysed using the framework method of thematic analysis [45] to facilitate the identification of commonalities and differences in data, and relationships between different parts of the data [46, 47]. This approach ensures that a priori issues (deductive approach) and themes that emerge from the adolescent’s narratives (inductive approach) guide the development of the analytic framework [48]. The qualitative analysis was carried out by two qualitative researchers. A codebook was developed that included ‘newly emerged’, ‘no change/stable’, ‘improved’, ‘worsened’ and ‘ceased/disappeared’ [37]. NVivo software was used to support the analysis. Data saturation was determined using a saturation grid [49] and was deemed to have been reached when no new concepts/subthemes had emerged.

## Results

Twenty-five adolescents enrolled into the MyXLH study: 10 from the UK, seven from France, four from Spain, three from the Netherlands and one from Germany. Nineteen (76%) continued burosumab after EoSG and six (24%) discontinued. Twenty-one adolescents were available for interview (15 who continued burosumab, 6 who discontinued). EoSG was mostly identified by growth velocity or imaging alone (*n* = 10 [40%] and *n* = 5 [20%], respectively), or the two methods combined with each other or with different methods (e.g. Tanner staging) (Table 1). EoSG occurred at mean age of 15.7 years in the sixteen girls and 17.3 years in the nine boys.

**Table 1:** Demographics, auxology, medical and treatment history at study enrolment in adolescents who continued and discontinued burosumab at EoSG

		Continued burosumab (*n* = 19)	Discontinued burosumab (*n* = 6)
Age at study enrolment (years), mean ± SD		15.2 ± 1.3	15.8 ± 1.5
Sex, n (%)	Female	14 (73.7)	2 (33.3)
	Male	5 (26.3)	4 (66.7)
Nationality, n(%)	UK	5 (26.3)	5 (83.3)
	France	7 (36.8)	0
	Spain	3 (15.8)	1 (16.7)
	Netherlands	3 (15.8)	0
	Germany	1 (5.3)	0
Standing height Z-score,^a^ mean ± SD	All adolescents	−0.9 ± 1.2	−0.8 ± 1.3
	Females	−0.8 ± 1.1	−1.3 ± 0.9
	Males	−1.1 ± 1.5	−0.5 ± 1.5
Weight (kg), mean ± SD	All adolescents	56.8 ± 14.8^b^	66.2 ± 9.6
	Females	52.6 ± 9.8	71.5 ± 12.0
	Males	71.5 ± 21.3	63.5 ± 8.7
Age at XLH diagnosis (years), mean ± SD		3.4 ± 3.2	1.8 ± 1.7
Age at EoSG (years), mean ± SD	All adolescents	16.1 ± 1.3	16.9 ± 1.4
	Females	15.8 ± 1.3	15.9 ± 1.4
	Males	17.2 ± 0.7	17.4 ± 1.2
EoSG assessment method, n (%)	Growth velocity	9 (47.4)	1 (16.7)
	Imaging	3 (15.8)	2 (33.3)
	Growth velocity and imaging	1 (5.3)	3 (50.0)
	Growth velocity, imaging, Tanner staging and other	2 (10.5)	0
	Imaging and other	1 (5.3)	0
	Other/not provided	3 (15.8)	0
Age at burosumab initiation (years), mean ± SD		11.8 ± 1.9	12.0 ± 1.6
Time on burosumab up to EoSG (years), mean ± SD		4.3 ± 1.9	4.9 ± 2.6

^a^Calculated using sex- and age-specific World Health Organization reference values [50]

^b^*n* = 18

EoSG, end of skeletal growth; SD, standard deviation; XLH, X-linked hypophosphataemia

### Adolescents who continued burosumab after EoSG

The 19 adolescents who continued burosumab treatment after EoSG were mostly from France (*n* = 7 [37%]) or the UK (*n* = 5 [26%]). The mean (SD) age at study enrolment was 15.2 (1.3) years; 74% of the adolescents were female. Mean (SD) standing height Z-score at study enrolment was −0.8 (1.1) in girls and −1.1 (1.5) in boys. Mean (SD) age at EoSG was 15.7 (1.3) years for girls and 17.2 (0.7) years for boys. Mean time on burosumab before EoSG was 4.3 (1.9) years (Table 1). The most common method for identifying EoSG in this group was growth velocity (*n* = 9 [47%]), followed by imaging (*n* = 3 [16%]).

#### Blood biochemistry

Blood biochemistry remained stable after EoSG (Table 2): mean (SD) serum phosphate concentration was 0.92 (0.21) mmol/L before EoSG and 0.88 (0.22) mmol/L after EoSG. Similar numbers of adolescents had serum phosphate levels in the normal range before and after EoSG (57% vs 56%). Mean (SD) ALP concentration was 154.1 (61.5) IU/L before EoSG and 144.4 (58.1) IU/L after EoSG. Fewer adolescents had normal ALP levels before compared with after EoSG (57% vs 63%). Mean (SD) PTH concentration was 5.9 (3.1) pmol/L before EoSG and 7.0 (4.9) after EoSG, with 86% of adolescents having normal PTH before EoSG and 77% after.

**Table 2:** Laboratory values, pain medication use and time missed from school/work before and after EoSG

			Continued burosumab (*n* = 19)				Discontinued burosumab (*n* = 6)			
			Before EoSG		After EoSG		Before EoSG		After EoSG	
			n	Value	n	Value	n	Value	n	Value
Serum biomarker concentrations, mean ± SD^a^	Phosphate (mmol/L)		14	0.92 ± 0.21	16	0.88 ± 0.22	3	0.86 ± 0.06	5	0.55 ± 0.12
	ALP (U/L)		14	154.1 ± 61.5	16	144.4 ± 58.1	3	253 ± 134.4	5	191.4 ± 108.0
	PTH (pmol/L)		14	5.9 ± 3.1	13	7.0 ± 4.9	3	5.1 ± 4.1	5	5.1 ± 1.7
Adolescents with normal^a^ serum biomarker values, n (%)^b^	Phosphate		14	8 (57.1)	16	9 (56.2)	3	2 (66.7)	5	0 (0.0)
	ALP		14	8 (57.1)	16	10 (62.5)	3	1 (33.3)	5	4 (80.0)
	PTH		14	12 (85.7)	13	10 (76.9)	3	1 (33.3)	5	4 (80.0)
Adolescents who reported using pain medication at least once, n (%)^c,d^			13^b^	8 (61.5)	5^b^	0 (0)^c^	5^b^	1 (20.0)	2^b^	1 (50.0)
Adolescents who reported time missed from work or school, n (%)			19	1 (5.3)	12	0 (0)^c^	5	1 (20.0)	3	0 (0.0)
EQ-5D-Y-3 L, mean ± SD		Utility score	15	0.86 ± 0.24	5	0.77 ± 0.30	5	0.94 ± 0.10	3	0.84 ± 0.15
		VAS		78.2 ± 17.6		78.4 ± 24.9		86.2 ± 7.9		85.0 ± 8.7

^a^Normalcy was reported by laboratories according to local reference ranges

^b^Laboratory tests performed mean 89.6 ± 60.6 (SD) days after EoSG for continued group and 94.4 ± 56.4 (SD) days for discontinued group

^c^Pain medication use reported only in those who reported pain

^d^Week 25 after EoSG

ALP, alkaline phosphatase; EoSG, end of skeletal growth; EQ-5D-Y-5 L, EuroQol five-dimension, three-level youth health survey; PTH, parathyroid hormone; SD, standard deviation; VAS, visual analogue scale

#### Symptoms

**Fig. 2:**
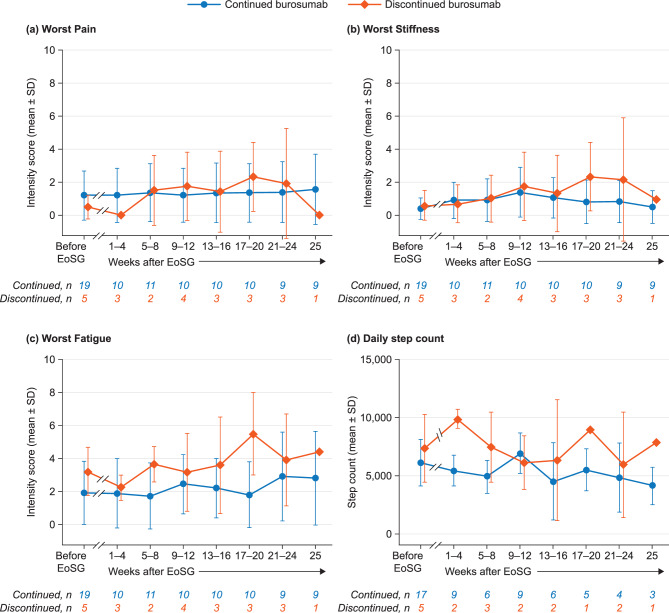
Symptoms and daily step count in adolescents before EoSG and for the 26 weeks following EoSG for adolescents who continued (*n* = 19) and discontinued (*n* = 6) burosumab. EoSG, end of skeletal growth; SD, standard deviation

Scores for Worst Fatigue, Worst Pain and Worst Stiffness (all measured on a 0–10 scale, with 10 indicating the worst problem) in the adolescents who continued burosumab treatment after EoSG are presented in Fig. 2. Data from the qualitative interviews provide contextual information for these scores (exemplar quotes provided in Table 3).

**Table 3:** Exemplar descriptions from the qualitative interviews in adolescents who continued on burosumab after EoSG (16 of 19 adolescents were interviewed)

	Worsened or newly emerging symptoms	No change, improved or ceased symptoms
Pain	“The discomfort bothers me, but lately it’s like it hurts a bit more. It bothers me more … I mean, after the surgeries, it began as just some discomfort. Now I’m feeling like pain, it’s rather pain and not some discomfort, especially on the left leg.”	“My legs and hips are actually bothering me less.” “Well, regarding the pain in the area of the legs and knees – what I already told you about 6 months ago – nothing really changed.” “… it is a bit less frequent.” *Interviewer: How often does it happen?* “Maybe once every 2 weeks, I think.”
Stiffness	“When I sat or laid down for a long time or did not do something for a long time, then I am very stiff in my legs and those kinds of things.” “It is a bit more frequent but, on the other hand, it is always in the same moment, in the evening when I have had a long day, in that moment I will have pain in my back, in the evening.”	“Here I’d say that it has consistently stayed the same. It still happens in the morning, and it only lasts for about 2–3 minutes. And generally speaking, nothing has changed.” *Interviewer: How often do you experience that stiffness?* “When I sit down usually. Every day.” *Interviewer: Is it daily?* “Yes.”
Fatigue	No one reported worsened or newly emerging fatigue	“No, no, it has not changed, if I sleep well, I don’t feel tired during the day.” “I’m tired after work, but other than that, I’m fine.” [laughs] “… sometimes when I come home from school, I’m tired, but simply because it’s been a long day.” “Yes, generally now I have more energy in the evenings as well as in the early morning.”
Sleep	“… sometimes my sleep schedules falls out a bit, it’s probably from stress though from like exams and stuff.”	“… I sleep better … I take around 45 minutes to fall asleep, but it is almost always like that, there is no 2 hours or things like that now.”
	*Interviewer: You talked about insomnia and nightmares that were quite regular as you said. Is it still the case? “*Yes. It is still the case, even more.”. *Interviewer: More nightmares or more insomnia or more insomnia because of the nightmares? “*Insomnia is every day and nightmares are usually every other night.”	“I think it’s better than it was 6 months ago.” *Interviewer*: *Okay. So, do you still wake up in the night?* “Not every night, sometimes.”
Physical activity	“… it is still the same. Even if I have pain or a bit of pain, I will do the activity.” *Interviewer: Are there any specific kind of activities that you find you can’t do when you have the pain?* “High im-, more high impact activities like running, or … yeah, long walk.” “Well, in the sense that it’s not that difficult for me to do most of things and that … how can I say it? If, for example, I struggled when running or doing something like that before, now I don’t find it that difficult.” “So, I can recall a few situations where I definitely managed to walk longer distances before the pain started.” *Interviewer: Do you still have trouble running because of the pain?* “Yes.” *Interviewer: And still trouble participating in gym class because of the pain?* “Yes.” “I’d say that when I’m out and about with my friends that I can keep up with them for longer, so to speak, before I need a break or something.”	
Emotions	“I’m happier that my leg doesn’t hurt.” “I think that it’s … okay, well. I think it is better, the stiffness bothers me less than the pain, therefore … no, no, it is okay, honestly … it makes me feel happy because I can [still] see that there is positive progress.” “Um, it bothers me, I mean, it bothers me quite a lot … I don’t know how to describe my feelings, but it’s like, I want it to stop and that’s it.” *Interviewer: And, as I say, in terms of emotions, you, you don’t feel anything, you know, the frustration and the annoyance, is that something you still have or has that gone completely?* “No, that’s gone.”	

In adolescents who continued burosumab, Worst Pain scores were low before EoSG and remained low over the 26 weeks after EoSG, with a mean score below 2 throughout. The mean (SD) score was 1.2 (1.5) before EoSG (*n* = 19) and ranged from 1.2 (1.6) at weeks 1–4 and 9–12 after EoSG (*n* = 10) to 1.6 (2.1) at week 25 (*n* = 9) (Fig. 2a). In the qualitative interviews (*n* = 15), five adolescents reported that their pain had not changed, in four it had improved and two reported cessation of pain. The remaining four described worsening pain in the time after EoSG, using terms such as sharpness, stabbing, nagging and achiness. Some of those who described worsening pain attributed this to a change in activities (e.g. being more active, sitting for a long time in exams).

Worst Stiffness scores were low before EoSG and remained low over the 26 weeks after EoSG, with a mean score below 2 throughout. The mean (SD) score was 0.4 (0.6) before EoSG (*n* = 19) and ranged from 0.5 (1.0) at week 25 after EoSG (*n* = 9) to 1.4 (1.5) at weeks 9–12 (*n* = 10) (Fig. 2b). In the qualitative interviews, most adolescents (13/15) reported no change in their stiffness in the time after EoSG, although eight of these had never experienced stiffness. Two adolescents described deteriorating or newly emerging stiffness.

Worst Fatigue scores were low before EoSG and remained low over the 26 weeks after EoSG, with a mean score below 3 throughout. The mean (SD) score was 1.9 (1.9) before EoSG (*n* = 19) and ranged from 1.7 (2.0) at weeks 5–8 after EoSG (*n* = 11) to 2.9 (2.7) at weeks 21–24 (*n* = 9) (Fig. 2c). In the qualitative interviews, 13 adolescents reported no change in their fatigue and two reported improvement. Fatigue was generally thought to be due to education and employment rather than XLH.

Quantitative sleep data were not captured in this study. In the qualitative interviews, most adolescents (10/15) reported no change in their sleep and three reported improvement. Two adolescents described worsening sleep in the time after EoSG, which one adolescent attributed to exams. Four adolescents reported experiencing light to moderate sleep problems (e.g. having difficulty sleeping two to three nights per week; having regular sleep interruptions during the night).

#### Physical activity

Data from the wearable device following EoSG were provided by nine adolescents for weeks 1–4, decreasing to three at week 25. For those who had data available, daily step count was broadly maintained (Fig. 2d). The activity diary data showed that the adolescents who continued with burosumab were exercising (e.g. walking, gym, climbing, dancing), doing sports (e.g. school sports, baseball), taking day trips (e.g. shopping, cinema, beach), going to school/college/work, and spending time with family and friends. In the qualitative interviews, some adolescents who described worsening of their pain since EoSG did not generally report any impact on their level of activity, whereas others reported that certain physical activities were affected (e.g. high-impact activities, long walks, running). Others described improved ability to do physical activities.

Survey data identified that one adolescent had missed time from work or school before EoSG, and after EoSG this ranged from 4 adolescents (weeks 1–4) to no adolescents (weeks 17–20 and 25) (Table 2).

#### HRQL

HRQL was measured before and after EoSG using the EQ-5D-Y-3 L; however, only 5/19 adolescents completed the measure after EoSG so quantitative data are limited (Table 2). Data from the qualitative interviews provide insight into the HRQL of the adolescents who continued burosumab treatment after EoSG. Example quotes relating to emotions are provided in Table 3.

The mean (SD) EQ-5D-Y-3 L utility score was 0.86 (0.24) before EoSG (*n* = 15) and 0.77 (0.30) after EoSG (*n* = 5). Few adolescents reported ‘some’ or ‘a lot’ of problems on the EQ-5D-Y-3 L items both before and after EoSG; the greatest problems were reported on the item measuring pain and discomfort (5/15 prior to EoSG, 3/5 after EoSG) (Supplementary Table 2).

In the qualitative interviews (*n* = 16), improved symptoms were linked to improved emotions (e.g. feeling happier, less frustration). One adolescent who experienced worsening of symptoms reported feeling bothered by them. Some adolescents referred to good and/or improved self-esteem and self-confidence. The adolescents described few impacts on their social relationships/activities and their education. Exemplar quotes are provided in Table 3.

#### Coping with XLH

In the qualitative interviews, adolescents described several strategies for coping with XLH symptoms and their impact; these included taking pain medication, using a hot tub, stretching, resting and talking with people. In the survey data, 62% of the adolescents reported using pain medication before EoSG and this ranged from 46% (weeks 1–4) to 0% (week 25) in the weeks after EoSG.

### Adolescents who discontinued burosumab after EoSG

Six adolescents discontinued burosumab after EoSG, five from the UK and one from Spain (Table 1); one adolescent subsequently restarted burosumab. Their mean (SD) age at study enrolment was 15.8 (1.5) years and 33% were female. Mean standing height Z-score was −1.3 (0.9) in girls and −0.5 (1.5) in boys. Mean age at EoSG was 17.4 (SD 1.2) years in boys and 15.9 (1.4) years in girls. Mean time on burosumab prior to EoSG was 4.9 (SD 2.6) years (Table 1).

Three of the six adolescents had blood biochemistry data available before EoSG and five after EoSG (Table 2). Serum phosphate levels were normal in 67% of the adolescents before EoSG and in none after EoSG. ALP was normal in 33% before EoSG and 80% after; PTH was also normal in 33% vs 80%. Individual biochemistry findings are presented in Supplementary Table 1.

Scores for Worst Fatigue, Worst Pain and Worst Stiffness (all measured on a 0–10 scale, with 10 indicating the worst problem) in the adolescents who discontinued burosumab treatment after EoSG are presented in Fig. 2, and individual scores for Worst Pain, Worst Stiffness and Worst Fatigue in Supplementary Figure 1. Following cessation of burosumab at EoSG, Worst Pain, Worst Stiffness and Worst Fatigue scores increased slightly but remained low over the 26 weeks after EoSG (Fig.s 2a–c). Data from the wearable device were limited following EoSG, ranging between three (weeks 5–8) and one (weeks 17–20, week 26) adolescents; for those who had data available, daily step count was broadly maintained (Fig. 2d).

The mean (SD) EQ-5D-Y-3 L utility score was 0.94 (0.10) before EoSG (*n* = 5) and 0.84 (0.15) after EoSG (*n* = 3). Group-level EQ-5D-Y-3 L scores are shown in Table 2 and individual EQ-5D-Y-3 L results in Supplementary Tables 2 and 3; exemplar quotes from the qualitative interviews are provided in Table 4 and Supplementary Table 4.

**Table 4:** Exemplar quotes from adolescents (*n* = 6) who stopped burosumab after EoSG

Symptom	Exemplar quotes
Pain	“Yeah, so my ankles really do hurt now after … So, this was never a, a problem before and on the … when I was on the injections, but now after I play football and you know, well move around a lot, my ankles really are sore and … but it, it’s like a, a bone pain. It just really hurts … I can ‘feel’ my hips, whereas before, when I was on the injections, yeah it was, it was pain … I don’t know, it ached a lot but then it was manageable.” “Yeah, I’m definitely experiencing some [pain]. Like, in my feet when I’m walking. Like, not for a long time, even when I just walk to uni for less than 10 minutes … Yeah, it’s just literally walking like, the first few steps I take out the door.” “Well, if I walk like, slowly then it’s not so bad, maybe like a 3 or a 4 [over 10] but if I … well, maybe like a 3. If I walk faster, then if I’m like, in a rush then it will be like a 6ish sometimes … it’s just painful like on my foot quite badly when I’m walking.” “It’s in my feet. Sometimes in my like, legs as well. Like … well, below the knee but mostly in the feet and it’s mostly in the right foot as well.” “And sometimes my chest as well just gets a bit … like the centre of my chest gets a bit like, I dunno [sic], painful. Like the … in the … not the heart or anything, but like in my ribs, if my … if I’m pushing it against something it just … yeah, hurts quite a bit.”
Stiffness	“Yeah, so it’s just kinda gone in like a direct positive correlation really. It’s … everything’s … like the pain, stiffness, it’s all increased a lot and yeah, it’s just … it’s a lot more prominent in my hips now and being stiff is, and my ankles as well, whereas it was, it was there before, but it wasn’t to the level it was now and like it eventually it would wear off throughout the day.” “The stiffness just … previously it was just in the morning whereas now it’s kind of into late afternoon before it frees up.” “I think my left knee is a little bit stiffer than my right knee and I have been kind of noticing that a little bit in the gym. It feels like I can’t move my left leg quite as much as my right sometimes but it’s not like an extreme amount, but it does definitely get annoying with some things and gets in the way.”
Fatigue	“Yeah, I think so fatigue as in like mental fatigue definitely has increased and physical fatigue as well, has increased.” “My main stiffness and pain’s gone up but the, the fatigue and tiredness hasn’t overly changed.” *Interviewer: So it does affect that movement that you need to do?* “Yes … Usually just drama, because I’m moving around, like all over.” *Interviewer: And so aside from interfering with your drama club, is there anything else that you find difficult or you can’t do because you’ve now got this pain?* “Um, no, not really; apart from like maybe bending down, but that’s it.” *Interviewer: OK, so it doesn’t affect like walking or you know, your daily activities?* “No.”
Physical activity	“I used to play three, three or four times a week, whereas now I only play once. Just because I, I can’t move [laughs].” “To give you an example. So before like I used to be able to run upstairs kind of thing, whereas now, it’s just no, I’m not even gonna [sic] try it, ‘cause [sic] I’ll probably end up falling down … ‘cause [sic] … yeah, and then my feet, yeah, ‘cause [sic] the stiffness is there a lot more so yeah, I just can’t move as fast in my legs, yeah.” “I do still like going, like walking, you know, travelling to … well, yeah, walking to places. I don’t … it’s not like I don’t wanna [sic] walk anywhere anymore. I still do … Yeah, I do enjoy walking, so it’s not really changed that. And the gym, it’s not really affected that either.”
Emotional state	“But it’s, yeah, I feel very … It’s very frustrating because the effect the injections had on my life is immeasurable, it’s been so … The, the difference now is insane between being on the injection and not being on the injection … That’s probably one of the most frustrating things in terms of not being able to have that injection to make the pain go, go away and prevent it from happening,’ cause [sic] yeah, obviously you get used to certain things and then them things are taken away and pain intensifies. It’s not nice … it’s more frustration at the fact I can’t have the, the injection, yeah. It’s not, yeah, it sounds a bit … Really, it’s not frustration in like in my … in my condition, or my legs. But it’s just more of a frustration now that there is something out there that has helped me a lot and will continue to help other people like that feel the same, but unfortunately, it’s just not licenced for adults, so yeah, it’s not really a frustration at myself anymore.” “So, like aside from being in pain obviously, it’s quite frustrating and ‘cause [sic] when, you know, when you need to, to like run, run for a train or something like that, you can’t, it’s like oh great. But yeah, it’s just frustrating really when you can’t move as you’d like to move.”

#### Adolescent 1

This male adolescent was diagnosed with XLH at 2.6 years of age. He started burosumab treatment at 10.7 years of age and continued until reaching EoSG (confirmed by growth velocity and imaging) at 18.2 years (7.4 years’ burosumab treatment). He then received oral phosphate supplementation and active vitamin D for 4 months.

Blood biochemistry assessments prior to EoSG were not recorded. Following termination of burosumab treatment at EoSG, serum phosphate was 0.40 mmol/L (below the lower limit of normal [LLN]), ALP was 128 IU/L (in normal range) and PTH was 3.46 pmol/L (in normal range).

Before EoSG, he had low scores for Worst Pain (mean [SD] 1.3 [1.3]), Worst Stiffness (1.9 [2.1]) and Worst Fatigue (1.9 [1.6]). Scores were not recorded after EoSG. In the qualitative interview, the adolescent reported worsened pain, stiffness and fatigue after stopping burosumab treatment at EoSG. He also described limitations to his physical activity because of worsening symptoms, including pain and stiffness affecting his work, and feeling frustrated. Before EoSG, he reported ‘no problems’ on all EQ-5D-Y-3 L items except for feeling ‘a bit worried, sad or unhappy’, with a utility score of 0.91. The EQ-5D-Y-3 L was not completed after EoSG. He reported no change in sleep quality following EoSG and he had not missed any time from school or work before EoSG or in the first 4 weeks after EoSG (no data were reported beyond the 4 weeks). No information was provided in his activity diary and mean daily step count was not available after EoSG.

Coping strategy options were felt to be limited, but included adapting how he lifted heavy objects at work, keeping moving to prevent stiffening, napping, going to bed early, and hiding symptoms at work by doing tasks that avoided walking. He reported using pain medication prior to EoSG and in the 4 weeks after EoSG but not in weeks 13–16 after EoSG (data were missing for the other periods after EoSG).

#### Adolescent 2

This female adolescent was diagnosed with XLH at 3.1 years of age. She started burosumab treatment at 12.6 years of age and continued until reaching EoSG at 14.9 years (confirmed by growth velocity and imaging) (2.3 years’ burosumab treatment). She subsequently started treatment with active vitamin D, which was ongoing at the time of the assessments.

Serum phosphate was 0.81 mmol/L before EoSG and 0.51 mmol/L after EoSG ( < LLN at both timepoints), ALP was 150 vs 175 IU/L (in normal range then at or above the upper limit of normal [ULN]) and PTH was 0.70 vs 6.30 pmol/L ( < LLN at both timepoints).

The mean (SD) score for Worst Pain and Worst Stiffness before EoSG was 0.1 (0.2), and 0 at weeks 1–4, 9–12, 13–16 and 21–24 after EoSG. The score for Worst Fatigue was 0.6 (1.0) before EoSG and steadily decreased to 0 at weeks 13–16 and 21–24 after EoSG. In the qualitative interview, the adolescent reported worsened pain, and no change in stiffness and fatigue after stopping burosumab treatment at EoSG. She also described limitations to her physical activity because of worsening symptoms. She described increased activity but did not connect this to her XLH treatment. She was happy she no longer needed to receive injections. Before EoSG, she reported ‘no problems’ on 4 out of 5 EQ-5D-Y-3 L items and ‘some problems’ for pain and discomfort; she had a utility score of 0.89. The EQ-5D-Y-3 L was not completed after EoSG. She reported no change in sleep quality following EoSG and reported missing half a day of work or school in weeks 21–24 after EoSG (data not provided for weeks 9–20 and 25 after EoSG). Activities reported in her activity diary included walking, cycling, swimming and sports day. Data from the wearable device were available for weeks 1–8 after EoSG; the mean daily step count was broadly maintained.

In the qualitative interview, she described sitting down to rest to help her cope with XLH symptoms. Although she reported ankle pain before EoSG and in weeks 17–20 after EoSG, she did not use pain medication (pain location and pain medication use were not reported at other timepoints post-EoSG).

#### Adolescent 3

This male adolescent was diagnosed with XLH at 0.2 years of age. He started burosumab treatment at 13.4 years of age and continued until reaching EoSG at 17.9 years (confirmed by growth velocity and imaging) (4.5 years’ burosumab treatment). He then received active vitamin D.

Blood biochemistry assessments prior to EoSG are not available. Following termination of burosumab treatment at EoSG, serum phosphate was 0.62 mmol/L ( < LLN), ALP was 150 IU/L (in normal range) and PTH was 6.20 pmol/L (in normal range).

During burosumab treatment he had low scores for Worst Pain (mean [SD] 1.6 [1.7]), Worst Stiffness (1.4 [1.1]) and Worst Fatigue 2.4 [1.4]). Scores increased at weeks 21–24 after EoSG to 5.6 (0.5), 6.3 (1.0) and 5.5 (1.3), respectively. In the qualitative interview, the adolescent reported worsened pain, stiffness and fatigue after stopping burosumab treatment at EoSG and less activity than before EoSG but felt that less activity was not due to worse symptoms. He reported ‘no problems’ on all EQ-5D-Y-3 L domains before EoSG, but after EoSG he reported ‘some problems’ on three domains (mobility, usual activities, having pain/discomfort). The EQ-5D-Y-3 L utility score was 1.00 before EoSG and 0.71 after EoSG. This adolescent reported no change in sleep quality following EoSG, and reported not missing any work or school days in weeks 1–20 after EoSG (no data were provided after week 20). Activities reported in his activity diary included walking, heavy lifting, badminton, table tennis and housework. Mean (SD) daily step count was 3449 (3122) before EoSG but was lower at weeks 5–8, 9–12, 13–16 and 21–24 after EoSG, ranging from 681 (84) at weeks 21–24 to 2601 (1831) at weeks 9–12.

Coping strategies included occasionally taking paracetamol when in pain, getting to bed earlier and sleeping a bit more. He reported not using any pain medication before EoSG but used pain medication on 4 days over the 26 weeks after EoSG.

#### Adolescent 4

This male adolescent was diagnosed with XLH at 0.2 years of age. He started burosumab treatment at 9.3 years of age and continued until reaching EoSG at 17.9 years (confirmed by growth velocity) (8.6 years’ burosumab treatment). Any subsequent treatment is not reported.

Before EoSG, serum phosphate was 0.92 mmol/L (in the normal range), ALP was 204 IU/L ( > ULN) and PTH was 6.00 pmol/L ( > ULN). Blood biochemistry assessments after EoSG are not available.

During burosumab treatment he had low scores for Worst Pain (mean [SD] 0.2 [0.4]), Worst Stiffness (0.4 [0.8]) and Worst Fatigue 3.8 [1.6]). Scores were not recorded after EoSG.

In the qualitative interview, the adolescent reported that pain and stiffness were newly emerged, and fatigue became worse after stopping burosumab treatment at EoSG. Foot pain was particularly prominent, even for short walks, and stiffness in one knee was an increasing problem. No subsequent changes in physical activity were described despite these changes in symptoms. Before EoSG, he reported ‘no problems’ on all EQ-5D-Y-3 L items, with a utility score of 1.00. The EQ-5D-Y-3 L was not completed after EoSG. This adolescent reported no change in sleep quality following EoSG, and reported not missing any days of work or school before EoSG or in weeks 1–4, 13–16 and 17–20 after EoSG when these data were collected. Activities reported in his activity diary included walking, gym, weight training and socialising with friends. Mean daily step count was not available after EoSG.

Coping strategies described included avoiding certain activities at the gym and resting. He reported no use of pain medication before EoSG or at weeks 5–8 after EoSG (the survey was not completed at the other timepoints).

#### Adolescent 5

This female adolescent was diagnosed with XLH at 0.8 years of age. She started burosumab treatment at 12.6 years of age and continued until reaching EoSG at 16.9 years (confirmed by imaging) (4.2 years’ burosumab treatment). She subsequently received oral phosphate supplementation and active vitamin D.

Blood biochemistry assessments before EoSG were not reported. Following termination of burosumab treatment at EoSG, serum phosphate was 0.49 mmol/L ( < LLN), ALP was 123 IU/L (in normal range) and PTH was 3.20 pmol/L (in normal range).

Pre-EoSG symptom scores were not reported. At weeks 1–4 after EoSG, mean (SD) scores were 0.6 (0.9) for Worst Pain, 2.2 (0.8) for Worst Stiffness and 1.2 (0.5) for Worst Fatigue, and increased to 4.0 (1.7), 3.7 (1.5) and 5.0 (1.0) respectively at weeks 17–20. In the qualitative interview, this adolescent reported newly emergent pain after stopping burosumab treatment at EoSG but no change in stiffness or fatigue. She described some consequential impact on physical activity but not on activities like walking. She described being content despite no longer receiving burosumab but also reported feeling annoyed about being unable to do things she used to do whilst receiving burosumab. She did not complete the EQ-5D-Y-3 L before EoSG. After EoSG, she reported ‘no problems’ on most domains but ‘some problems’ with pain/discomfort and feeling ‘a bit worried, sad or unhappy’. Her post-EoSG utility score was 0.83. She reported no change in sleep quality following EoSG, and did not miss any work or school days after EoSG. No information was provided in her activity diary and mean daily step count was not available after EoSG.

No coping strategies were described and no data on pain medication use after EoSG were reported.

#### Adolescent 6

This male adolescent was diagnosed with XLH at 4.1 years of age. He started burosumab treatment at 13.2 years of age and continued until reaching EoSG at 15.6 years (confirmed by imaging) (2.3 years’ burosumab treatment). He then received active vitamin D but returned to burosumab treatment 79 days later.

EoSG biochemistry assessments were taken 12 days after restarting burosumab treatment. Serum phosphate was 0.84 mmol/L (in the normal range) before EoSG and 0.71 mmol/L ( < LLN) after EoSG. ALP was 405 and 381 IU/L, respectively ( > ULN before EoSG, in normal range after), and PTH was 8.71 and 6.51 pmol, respectively ( > ULN at both timepoints).

Before EoSG this adolescent had low scores for Worst Pain (mean [SD] 0.2 [0.6]), Worst Stiffness (0.2 [0.4]) and Worst Fatigue (1.4 [1.1]). Worst Pain scores were highest in weeks 9–12 after EoSG (0.3 [0.8]) when off burosumab treatment, and decreased to 0 for weeks 17–25, during which time (week 20) he resumed burosumab treatment. However, Worst Stiffness scores declined steadily to 0.9 in week 25 after EoSG, and Worst Fatigue scores varied from 0.50 (weeks 9–12) to 3.0 (week 25) in the weeks after EoSG. In the qualitative interview, this adolescent reported less wrist pain and no changes in knee pain, stiffness or fatigue during the time when burosumab treatment had stopped at EoSG. After returning to burosumab treatment, knee pain was described as having ceased, with no change in stiffness or fatigue. He reported no change to his emotional state following EoSG during the period when he had stopped burosumab or after resuming treatment, except for feeling relieved that the knee pain had stopped. Before EoSG, he reported ‘no problems’ on any EQ-5D-Y-3 L domain except for feeling ‘a bit worried, sad or unhappy’ and reported ‘no problems’ on all domains after EoSG. His utility scores were 0.91 before EoSG and 1.00 after EoSG. He missed half a day of work or school before EoSG and half a day each in weeks 9–12 and 13–16 after EoSG. Activities recorded in his activity diary included music, football, dancing, and going out with friends. Mean (SD) daily step count decreased from 8680 (3655) before EoSG to 6755 (3963) at week 25.

This adolescent did not describe any coping strategies and did not use pain medication before or after EoSG.

### Safety results

A total of 86 adverse events and 14 special situations, all non-serious, were reported during the study. Eight events were considered to be related to burosumab: two events reported by the study investigator and a further six events assessed by the sponsor, comprising three cases of pain in an extremity and one case each of joint stiffness, arthralgia, injection site pain, back pain and nausea.

## Discussion

This mixed-methods observational study of adolescents with XLH treated with burosumab indicates that discontinuing burosumab at EoSG may be detrimental for adolescents with XLH. While the collection of patient-reported outcome scores was incomplete, in one-to-one interviews, adolescents who discontinued burosumab at EoSG reported worsening or newly emergent pain, fatigue and stiffness, and negative effects on physical and social activities, employment and emotional/mental state. These findings are consistent with two recently reported case series. In a descriptive case series of eight adolescents who stopped burosumab treatment at EoSG (at ages 13.2–18.5 years; median duration of treatment 37.5 months [range 22–53]), patients reported worsening or return of pain, decreased physical function, increased tiredness, reduced participation in sport, work, school and social activities, and a marked detriment in mental wellbeing, especially as their symptoms had improved while receiving burosumab [22]. A case series of five adolescents (mean age 15.4 ± 1.5 years) who received burosumab treatment until 18 or 19 years of age (12–48 months’ treatment) reported that discontinuation of burosumab treatment in four patients was associated with a decrease in serum phosphate, reversal of other improvements in clinical chemistry, and progressive worsening of patient-reported pain, stiffness and physical function [23]. By contrast, adolescents in the current study who continued burosumab treatment beyond EoSG had stable bone biochemistry markers, stiffness and fatigue scores were low and stable, and pain levels were stable or improved. In the interviews, these adolescents talked about improved wellbeing and little impact of symptoms on social and physical activities and education. A 2022 consensus statement highlighted the importance of considering the social and psychological, as well as physical, impacts of XLH [51].

Growth curves for paediatric patients in the International XLH Registry, 46% of whom were receiving burosumab treatment at the time of the assessments, showed that Z-scores consistently tracked at approximately −2 from 1 year of age onwards throughout life [52]. In the current study, the mean (SD) standing height Z-score at study enrolment was −0.8 (1.1) in girls and −1.1 (1.5) in boys, indicating that this sample of adolescents receiving burosumab were closer to the World Health Organization normal reference values for height than the previously reported sample.

The need for continued treatment to maintain the benefits of burosumab has been demonstrated in a recent study in adults – an exploratory analysis based on seven patients whose treatment was interrupted for up to 15 months found that some of the benefits of burosumab treatment were lost during this period; however, benefits returned over 36 weeks or longer when treatment resumed [24]. This is consistent with the evidence from the current study and previous case series [22, 23] that the benefits of burosumab treatment are lost when treatment is stopped; this is also consistent with the mechanism of action of burosumab in inhibiting the excess FGF23 activity that characterises XLH.

Clinical practice guidelines on the management of XLH suggest that continuation of burosumab for at least several years after growth plate closure could optimise bone accrual and mineralisation [19, 20]. In addition, osteomalacia of XLH may persist or recur in adulthood even if treated during childhood [19]. The findings of the current study and the two case series are consistent with this recommendation, indicating that symptomatic and biochemical improvements and HRQL are maintained with continued burosumab treatment, although specific effects on bone morphometry have yet to be explored. Ideally, the impact of stopping burosumab at EoSG and either resuming treatment or not as an adult would be explored in clinical studies.

Whilst clinical guidelines recommend continued treatment, and burosumab has marketing approval in more than 30 countries, including in the EU, US, Japan and Canada, local funding/reimbursement varies across jurisdictions, and in some European countries, adolescents stop treatment at EoSG. In the current study, EoSG occurred earlier in girls than boys (at a mean age of 15.7 years in the 16 girls and 17.3 years in the 9 boys). Jarvis and colleagues reported a mean age at end of treatment (i.e., at EoSG) of 15.0 years in the five girls (range 13.2–18.5) and 17.1 years (17.3–17.8) in the three boys. Depending on the criterion used for EoSG, girls may stop burosumab treatment earlier than boys, potentially facing increased pain, worse mobility and compromised mental health, both at a younger age and for longer than boys [22].

Real-world data are increasingly valued by regulators and payers to understand the natural history of rare diseases [53]. The current study used mixed quantitative–qualitative methodology, which overcomes some of the challenges encountered when researching rare diseases [54] and is endorsed by the International Rare Disease Research Consortium as the ‘best fit’ for rare diseases [37]. Inferences drawn from the combination of quantitative and qualitative data provide greater insight and learning than either data source alone, and also provide a patient-centric view [54]. The mixed-methods approach, combining group-level analysis and individual summaries for the six adolescents who stopped burosumab, facilitated the reporting of results for a small, heterogeneous real-world sample with evidence from multiple sources, and mitigated potential misinterpretations from missing data in group-level reporting. The one-to-one interviews in the current study gave important insight into the impact of the physical symptoms of XLH on daily activities, education and sleep, and were particularly insightful for the adolescents who stopped burosumab at EoSG, as data were insufficient to allow group-level analysis. Use of digital technology for real-world data collection is considered more representative of free-living conditions than an in-clinic snapshot of the participants’ physiology, behaviour and function that may be prone to biases and artifacts [55].

Limitations include the substantial missing quantitative data, which is a common issue in real-world studies [56]. Although the study was co-designed by patients, who considered the use of a wearable device and a bespoke app to be appropriate [27], in reality, data reporting was lower than expected, particularly from the wearable device, even though adolescents received prompts and notifications. This finding is not unique to this study or to studies in adolescents [57, 58]. The reasons for this need to be understood and strategies developed to improve engagement and data collection in future studies. These might include providing immediate data feedback from the wearable device, displaying the percentage completion of data collection, and use of reward-based systems. Use of participants’ own devices, which was the case for most adolescents in this study, is expected to yield better compliance with data collection than a study-provided device [55]. Conducting research in adolescents can be challenging when they are experiencing many changes, such as puberty, environmental changes (e.g. rising school workload), and less physical activity [59]. Clinical data were collected retrospectively from medical records and therefore timing and completeness could not be guaranteed. Local reference ranges were used to interpret the normality of blood biochemistry values, which may have led to wider variation than in a clinical trial setting. The timing of assessments in the dosing cycle was not standardized and may also have led to wider variation than in a trial setting. There is no consensus on how EoSG is defined across study centres or countries. In this study EoSG was identified by clinicians using local methods – largely growth velocity or imaging. The relationship between diet and vitamin D intake was not assessed in this study. Extending the follow-up period beyond 26 weeks would provide more comprehensive evidence of the impact of continuing or discontinuing burosumab after EoSG.

It is hoped that evidence from this mixed-methods study adds to the literature for those counselling adolescents and their parents or care partners for ongoing burosumab treatment, and aids healthcare systems to individualize care in XLH. Further real-world data from adolescents who receive continuous treatment with burosumab may provide a useful comparison with those whose treatment is stopped or interrupted, and also contribute to the evidence base for burosumab treatment in adolescents with XLH. Real-world data are currently being collected in the International XLH Registry (NCT03193476 [52, 60]; XLH Disease Monitoring Program (NCT03651505) and SUNFLOWER (NCT03745521).

## Conclusion

Stopping burosumab treatment at EoSG led to decreased serum phosphate concentrations and in some patients was associated with worsening of functional XLH symptoms and negative impacts on broad aspects of life. Symptom control varied between patients. By contrast, adolescents who continued burosumab after EoSG maintained serum phosphate concentrations and symptom control. This suggests that continuing burosumab is likely to be beneficial, but further investigation is needed, particularly for adolescents who stop burosumab at EoSG.

## Electronic supplementary material

Below is the link to the electronic supplementary material.

Supplementary Material 1

## Data Availability

Data that underlie the results reported in this article may be requested. Kyowa Kirin International will review requests individually to determine whether requests are legitimate, relevant and meet sound scientific principles, and are within the scope of the participants’ informed consent.

## Abbreviations

ALP: Alkaline phosphatase
EoSG: End of skeletal growth
EQ-5D-Y-3 L: EuroQol 5-dimension, 3-level youth health survey
FGF23: Fibroblast growth factor 23
HRQL: Health-related quality of life
LLN: Lower limit of normal
PTH: Parathyroid hormone
SD: Standard deviation
ULN: Upper limit of normal
XLH: X-linked hypophosphataemia

## References

[1] Beck-Nielsen SS, Mughal Z, Haffner D, et al. FGF23 and its role in X-linked hypophosphatemia-related morbidity. Orphanet J Rare Dis. 2019;14(1):58. 30808384 10.1186/s13023-019-1014-8 PMC6390548

[2] Linglart A, Biosse-Duplan M, Briot K, et al. Therapeutic management of hypophosphatemic rickets from infancy to adulthood. Endocr Connect. 2014;3(1):R13–30. 10.1530/EC-13-0103 PMC3959730 24550322

[3] Collins M. Burosumab: at long last, an effective treatment for FGF23-associated hypophosphatemia. J Bone Min Res. 2018;33(8):1381–82. 10.1002/jbmr.3544 29989668

[4] Skrinar A, Dvorak-Ewell M, Evins A, et al. The lifelong impact of X-linked hypophosphatemia: results from a burden of disease survey. J Endocr Soc. 2019;3(7):1321–34. 31259293 10.1210/js.2018-00365 PMC6595532

[5] Chen YY, Kao TW, Chou CW, et al. Exploring the link between serum phosphate levels and low muscle strength, dynapenia, and sarcopenia. Sci Rep. 2018;8(1):3573. 29476104 10.1038/s41598-018-21784-1 PMC5824959

[6] Veilleux LN, Cheung M, Ben Amor M, Rauch F. Abnormalities in muscle density and muscle function in hypophosphatemic rickets. J Clin Endocrinol Metab. 2012;97(8):E1492–8. 10.1210/jc.2012-1336 22639288

[7] Cremonesi I, Nucci C, D’Alessandro G, et al. X-linked hypophosphatemic rickets: enamel abnormalities and oral clinical findings. Scanning. 2014;36(4):456–61. 24677288 10.1002/sca.21141

[8] Brandi ML, Jan de Beur S, Briot K, et al. Efficacy of burosumab in adults with X-linked hypophosphatemia (XLH): a post hoc subgroup analysis of a randomized double-blind placebo-controlled phase 3 study. Calcif Tissue Int. 2022;111(4):409–18. 35927518 10.1007/s00223-022-01006-7

[9] Seefried L, Alzahrani A, Arango Sancho P, et al. XLH matters, 2022: insights and recommendations to improve outcomes for people living with X-linked hypophosphataemia (XLH). Orphanet J Rare Dis. 2023;18(Suppl 2):333. 37885021 10.1186/s13023-023-02883-3 PMC10604503

[10] Kamenicky P, Briot K, Munns CF, Linglart A. X-linked hypophosphataemia. Lancet. 2024;404(10455):887–901. 39181153 10.1016/S0140-6736(24)01305-9

[11] Ferizovic N, Marshall J, Williams AE, et al. Exploring the burden of X-linked hypophosphataemia: an opportunistic qualitative study of patient statements generated during a technology appraisal. Adv Ther. 2020;37(2):770–84. 31865548 10.1007/s12325-019-01193-0 PMC7004427

[12] Cheung M, Rylands AJ, Williams A, et al. Patient-reported complications, symptoms, and experiences of living with X-linked hypophosphatemia across the life-course. J Endocr Soc. 2021;5(8):bvab070. 10.1210/jendso/bvab070 PMC8272533 34258488

[13] Linglart A, Dvorak-Ewell M, Marshall A, San Martin J, Skrinar A. Impaired mobility and pain significantly impact the quality of life of children with X-linked hypophosphatemia. Bone Abstr. 2015;4.

[14] Linglart A, Amouroux C, Gueorguieva I, et al. Health-related quality of life in French paediatric patients with X-linked hypophosphataemia: real-world data from the International XLH Registry JBMR Plus. 2025, In press. 10.1093/jbmrpl/ziaf142 PMC12478027 41030383

[15] Hamdy NAT, Harvengt P, Usardi A. X-linked hypophosphatemia: the medical expert’s challenges and the patient’s concerns on their journey with the disease. Arch Pediatr. 2021;28(7):612–18. 34593293 10.1016/j.arcped.2021.09.005

[16] Insogna KL, Rauch F, Kamenicky P, et al. Burosumab improved histomorphometric measures of osteomalacia in adults with X-linked hypophosphatemia: a phase 3, single-arm, international trial. J Bone Min Res. 2019;34(12):2183–91. 10.1002/jbmr.3843 PMC6916280 31369697

[17] Imel EA, Glorieux FH, Whyte MP, et al. Burosumab versus conventional therapy in children with X-linked hypophosphataemia: a randomised, active-controlled, open-label, phase 3 trial. Lancet. 2019;393(10189):2416–27. 31104833 10.1016/S0140-6736(19)30654-3 PMC7179969

[18] Padidela R, Whyte MP, Glorieux FH, et al. Patient-reported outcomes from a randomized, active-controlled, open-label, phase 3 trial of burosumab versus conventional therapy in children with X-linked hypophosphatemia. Calcif Tissue Int. 2021;108(5):622–33. 33484279 10.1007/s00223-020-00797-x PMC8064984

[19] Ali DS, Carpenter TO, Imel EA, et al. X-Linked hypophosphatemia management in children: an international working group clinical practice guideline. J Clin Endocrinol Metab. 2025;110:2055–70. 39960858 10.1210/clinem/dgaf093 PMC12187519

[20] Haffner D, Emma F, Seefried L, et al. Clinical practice recommendations for the diagnosis and management of X-linked hypophosphataemia. Nat Rev Nephrol. 2025;21(5):330–54. 39814982 10.1038/s41581-024-00926-x

[21] Mughal MZ, Baroncelli GI, de Lucas-Collantes C, et al. Burosumab for X-linked hypophosphatemia in children and adolescents: opinion based on early experience in seven European countries. Front Endocrinol (Lausanne). 2023;13:1034580. 36798486 10.3389/fendo.2022.1034580 PMC9928183

[22] Jarvis C, Ramakrishnan R, Dharmaraj P, et al. Impact of stopping burosumab treatment at the end of skeletal growth in adolescents with X-linked hypophosphatemia (XLH). Bone Rep. 2025;24:101819. 39679164 10.1016/j.bonr.2024.101819 PMC11638637

[23] Baroncelli GI, Grandone A, Aversa A, et al. Safety and efficacy of burosumab in improving phosphate metabolism, bone health, and quality of life in adolescents with X-linked hypophosphatemic rickets. Eur J Med Genet. 2024;70:104958. 38950880 10.1016/j.ejmg.2024.104958

[24] Kamenicky P, Briot K, Brandi ML, et al. Benefit of burosumab in adults with X-linked hypophosphataemia (XLH) is maintained with long-term treatment. RMD Open. 2023;9(1). 10.1136/rmdopen-2022-002676 PMC9980374 36854566

[25] Boot A, Carpenter T, Högler W, et al. Benefits of long-term burosumab persist in 11 girls with X-linked hypophosphatemia (XLH) who transitioned into adolescence during the phase 2 CL201 trial. ESPE Abstr. 2019;92: FC2.

[26] Saraff V, Arango Sancho P, Bacchetta J, et al. Health related quality of life (HRQoL) of adolescents with XLH treated with burosumab at the end of skeletal growth (EoSG). ESPE Abstr. 2024;98:P1–116.

[27] Saraff V, Boot AM, Linglart A, et al. A patient-centred and multi-stakeholder co-designed observational prospective study protocol: example of the adolescent experience of treatment for X-linked hypophosphataemia (XLH). PLoS One. 2024;19(1):e0295080. 10.1371/journal.pone.0295080 PMC10798437 38241270

[28] Saraff V, Sancho P, Bacchetta J, et al. Adolescents with X-linked hypophosphatemia treated with burosumab at end of skeletal growth: a mixed-methods analysis. PLoS One. 2026. 10.1371/journal.pone.0344902 PMC13004326 41860923

[29] STROBE Initiative. STROBE statement: available checklists [Internet]. 2009. Available from: https://www.strobe-statement.org/index.php?id=available-checklists.

[30] Equator Network. Consolidated criteria for reporting qualitative research (COREQ): a 32-item checklist for interviews and focus groups. 2023. Available from: https://www.equator-network.org/reporting-guidelines/coreq/. 10.1093/intqhc/mzm042 17872937

[31] EuroQol. EQ-5D-Y-3L. 2009. Available from: https://euroqol.org/information-and-support/euroqol-instruments/eq-5d-y-3l/.

[32] Calman L, Brunton L, Molassiotis A. Developing longitudinal qualitative designs: lessons learned and recommendations for health services research. BMC Med Res Methodol. 2013;13(1):14. 23388075 10.1186/1471-2288-13-14 PMC3598728

[33] Astbury NM, Albury C, Nourse R, Jebb SA. Participant experiences of a low-energy total diet replacement programme: a descriptive qualitative study. PLoS One. 2020;15(9):e0238645. 10.1371/journal.pone.0238645 PMC7478843 32898176

[34] Bradshaw C, Atkinson S, Doody O. Employing a qualitative descriptive approach in health care research. Glob Qual Nurs Res. 2017;4:1–8. 10.1177/2333393617742282 PMC5703087 29204457

[35] Morse JM. Data were saturated…. Qual Health Res. 2015;25(5):587–88. 25829508 10.1177/1049732315576699

[36] European Medicines Agency. Guideline on good pharmacovigilance practices (GVP) module VI - Collection, management and submission of reports of suspected adverse reactions to medicinal products (Rev 2). Internet. 2017. Available from: http://www.ema.europa.eu/docs/en_GB/document_library/Regulatory_and_procedural_guideline/2017/08/WC500232767.pdf.

[37] Bharmal M, Guillemin I, Marrel A, et al. How to address the challenges of evaluating treatment benefits-risks in rare diseases? A convergent mixed methods approach applied within a merkel cell carcinoma phase 2 clinical trial. Orphanet J Rare Dis. 2018;13(1):95. 29914528 10.1186/s13023-018-0835-1 PMC6006962

[38] Day J, Jayatilleke C, Roy M. Improvements with burosumab treatment in an early access program for adults with X-linked hypophosphataemia: a case series of three patients. Bone Rep. 2024, In press. 10.1016/j.bonr.2024.101814 PMC11609550 39624115

[39] Jan de Beur SM, Cimms T, Nixon A, et al. Burosumab improves patient-reported outcomes in adults with tumor-induced osteomalacia: mixed-methods analysis. J Bone Min Res. 2023. 10.1002/jbmr.4900 37578099

[40] Creswell J, Plano-Clark V. Designing and conducting mixed methods research. 3rd ed. London: Sage; 2017.

[41] Cadiou S, Chapurlat R, Couture G, et al. Real-world efficacy and safety of burosumab in tumor-induced osteomalacia: case series from an early access program. JBMR Plus. 2025;9(6):ziaf039. 10.1093/jbmrpl/ziaf039 PMC12050030 40329993

[42] Mindler GT, Stauffer A, Kranzl A, et al. Persistent lower limb deformities despite amelioration of rickets in X-linked hypophosphatemia (XLH) - a prospective observational study. Front Endocrinol (Lausanne). 2022;13:866170. 35399930 10.3389/fendo.2022.866170 PMC8987359

[43] Ramos-Goni JM, Oppe M, Estevez-Carrillo A, et al. Accounting for unobservable preference heterogeneity and evaluating alternative anchoring approaches to estimate country-specific EQ-5D-Y value sets: a case study using Spanish preference data. Value Health. 2022;25(5):835–43. 35500952 10.1016/j.jval.2021.10.013

[44] Morton F, Singh Nijjar J. EQ-5D: methods for analysing ‘EQ-5D’ data and calculating ‘EQ-5D’ index scores. 2025. Available from: https://cran.r-project.org/web/packages/eq5d/index.html.

[45] Ritchie J, Lewis J, McNaughton Nicholls C, Ormston R. Qualitative research practice: a guide for social science students. London: Sage Publications; 2014.

[46] Gale NK, Heath G, Cameron E, et al. Using the framework method for the analysis of qualitative data in multi-disciplinary health research. BMC Med Res Methodol. 2013;13:117. 24047204 10.1186/1471-2288-13-117 PMC3848812

[47] Njau B, Lisasi E, Damian DJ, et al. Feasibility of an HIV self-testing intervention: a formative qualitative study among individuals, community leaders, and HIV testing experts in northern Tanzania. BMC Public Health. 2020;20(1):490. 32293370 10.1186/s12889-020-08651-3 PMC7161285

[48] Parkinson S, Eatough V, Holmes J, et al. Framework analysis: a worked example of a study exploring young people’s experiences of depression. Qual Res Psychol. 2016;13(2):109–29.

[49] Brod M, Tesler LE, Christensen TL. Qualitative research and content validity: developing best practices based on science and experience. Qual Life Res. 2009;18:1263–78. 19784865 10.1007/s11136-009-9540-9

[50] WHO. Growth reference data, height-for-age (5-19 years). 2025. Available from: https://www.who.int/tools/growth-reference-data-for-5to19-years/indicators/height-for-age.

[51] Trombetti A, Al-Daghri N, Brandi ML, et al. Interdisciplinary management of FGF23-related phosphate wasting syndromes: a consensus statement on the evaluation, diagnosis and care of patients with X-linked hypophosphataemia. Nat Rev Endocrinol. 2022;18(6):366–84. 35484227 10.1038/s41574-022-00662-x

[52] Ariceta G, Beck-Nielsen SS, Boot AM, et al. The International X-Linked hypophosphatemia (XLH) Registry: first interim analysis of baseline demographic, genetic and clinical data. Orphanet J Rare Dis. 2023;18(1):304. 37752558 10.1186/s13023-023-02882-4 PMC10523658

[53] Graili P, Guertin JR, Chan KKW, Tadrous M. Integration of real-world evidence from different data sources in health technology assessment. J Pharm Pharm Sci. 2023;26:11460. 37529633 10.3389/jpps.2023.11460 PMC10387532

[54] Morel T, Cano SJ. Measuring what matters to rare disease patients - reflections on the work by the IRDiRC taskforce on patient-centered outcome measures. Orphanet J Rare Dis. 2017;12(1):171. 29096663 10.1186/s13023-017-0718-x PMC5667521

[55] Demanuele C, Lokker C, Jhaveri K, et al. Considerations for conducting bring your own “device” (BYOD) clinical studies. Digit Biomark. 2022;6(2):47–60. 35949223 10.1159/000525080 PMC9294934

[56] Yang D, Miccio A, Jairam V, et al. The impact of missing/incomplete data in real-world data studies. Int J Radiat Oncol Biol Phys. 2020;108(3):E394.

[57] Schaefer S, Carter Ching C, Breen H, German J. Wearing, thinking, and moving: testing the feasibility of fitness tracking with urban youth. Am J Health Ed. 2016;47(1):8–16.

[58] Chandrasekaran R, Sadiq TM, Moustakas E. Usage trends and data sharing practices of healthcare wearable devices among US adults: cross-sectional study. J Med Internet Res. 2025;27:e63879. 10.2196/63879 PMC11890132 39982763

[59] Chen X, Wang F, Zhang H, et al. Effectiveness of wearable activity trackers on physical activity among adolescents in school-based settings: a systematic review and meta-analysis. BMC Public Health. 2025;25(1):1050. 40102761 10.1186/s12889-025-22170-z PMC11921619

[60] Padidela R, Nilsson O, Makitie O, et al. The international X-linked hypophosphataemia (XLH) registry (NCT03193476): rationale for and description of an international, observational study. Orphanet J Rare Dis. 2020;15(1):172. 32605590 10.1186/s13023-020-01434-4 PMC7329472

