# Supplementary material for: Adolescents’ experience of living with X-linked hypophosphataemia (XLH): a mixed-methods analysis of those who continued and discontinued burosumab treatment after end of skeletal growth
Source: Orphanet J Rare Dis. 2026 Feb 3;21:107. doi: 10.1186/s13023-026-04244-2 (PMC13020182; doi:10.1186/s13023-026-04244-2)
Supplement: Supplementary file 1 — Supplementary Material 1 [file 13023_2026_4244_MOESM1_ESM.docx]

# Supplementary tables and figures

Supplementary Table 1 Biochemistry values in adolescents who stopped burosumab treatment at EoSG

| Adolescent | Phosphate (mmol/L) | | ALP (IU/L) | | PTH (pmol/L) | |
| --- | --- | --- | --- | --- | --- | --- |
|  | **Before EoSG** | **After EoSG^a^** | **Before EoSG** | **After EoSG ^a^** | **Before EoSG** | **After EoSG ^a^** |
| 1 | NR | 0.40 | NR | 128 | NR | 3.46 |
| 2 | 0.81 | 0.51 | 150 | 175 | 0.70 | 6.30 |
| 3 | NR | 0.62 | NR | 150 | NR | 6.20 |
| 4 | 0.92 | NR | 204 | NR | 6.00 | NR |
| 5 | NR | 0.49 | NR | 123 | NR | 3.20 |
| 6^b^ | 0.84 | 0.71 | 405 | 381 | 8.71 | 6.51 |
| ^a^Laboratory tests taken mean 94.4 ± 56.4 (SD) days after EoSG  ^b^Stopped burosumab for 79 days, restarted burosumab 12 days prior to the EoSG assessments  ALP, alkaline phosphatase; EoSG, end of skeletal growth; NR, not recorded; PTH, parathyroid hormone | | | | | | |

Supplementary Table 2 EQ-5D-Y-3L domain responses before and after EoSG for adolescents who continued and discontinued burosumab

| Domain | Extent of problems | Continued burosumab | | Discontinued burosumab | |
| --- | --- | --- | --- | --- | --- |
|  |  | **Before EoSG (n=15)** | **After EoSG (n=5)** | **Before EoSG (n=5)** | **After EoSG (n=3)** |
| Mobility | None | 13 (86.7) | 3 (60.0) | 5 (100.0) | 2 (66.7) |
|  | Some | 2 (13.3) | 2 (40.0) | 0 (0) | 1 (33.3) |
|  | A lot | 0 (0) | 0 (0) | 0 (0) | 0 (0) |
| Looking after myself | None | 13 (86.7) | 5 (100.0) | 5 (100.0) | 3 (100.0) |
|  | Some | 2 (13.3) | 0 (0) | 0 (0) | 0 (0) |
|  | A lot | 0 (0) | 0 (0) | 0 (0) | 0 (0) |
| Usual activities | None | 15 (100.0) | 3 (60.0) | 5 (100.0) | 2 (66.7) |
|  | Some | 0 (0) | 2 (40.0) | 0 (0) | 1 (33.3) |
|  | A lot | 0 (0) | 0 (0) | 0 (0) | 0 (0) |
| Pain/discomfort | None | 10 (66.7) | 2 (40.0) | 4 (80.0) | 1 (33.3) |
|  | Some | 5 (33.3) | 3 (60.0) | 1 (20.0) | 2 (66.7) |
|  | A lot | 0 (0) | 0 (0) | 0 (0) | 0 (0) |
| Worried, sad or unhappy | None | 10 (66.7) | 4 (80.0) | 3 (60.0) | 2 (66.7) |
|  | Some | 3 (20.0) | 0 (0) | 2 (40.0) | 1 (33.3) |
|  | A lot | 2 (13.3) | 1 (20.0) | 0 (0) | 0 (0) |
| Values are n (%) of respondents who continued burosumab; 15/19 adolescents who continued burosumab and 5/6 who discontinued burosumab completed the EQ-5D-Y-3L  EoSG, end of skeletal growth; EQ-5D-Y-3L, EuroQol five-dimension, three-level youth health survey | | | | | |

Supplementary Table 3 EQ-5D-Y-3L utility scores in adolescents who stopped burosumab treatment at EoSG

| Adolescent | Utility score | |
| --- | --- | --- |
|  | **Before EoSG** | **After EoSG** |
| 1 | 0.91 | NR |
| 2 | 0.89 | NR |
| 3 | 1.00 | 0.71 |
| 4 | 1.00 | NR |
| 5 | NR | 0.83 |
| 6^a^ | 0.91 | 1.00 |
| ^a^Stopped burosumab for 79 days and then restarted  EoSG, end of skeletal growth; EQ-5D-Y-3L, EuroQol five-dimension, three-level youth health survey; NR, not recorded | | |

Supplementary Table 4 Exemplar quotes from the six adolescents who stopped burosumab after end of skeletal growth

| Adolescent | Exemplar quotes |
| --- | --- |
| **Pain** | |
| 1 | “Yeah, so my ankles really do hurt now after … So, this was never a, a problem before and on the … when I was on the injections, but now after I play football and you know, well move around a lot, my ankles really are sore and … but it, it’s like a, a bone pain. It just really hurts … I can ‘feel’ my hips, whereas before, when I was on the injections, yeah it was, it was pain … I don’t know, it ached a lot but then it was manageable.” |
| 2 | “Well, since … I don’t get much pain unless I’ve been standing up for a while.”  “Most of the time, I feel no pain.”  “I think the ankle pain is more often.”  “I would say that the, the ankle pain is only caused when I’m like walking for a long time.” |
| 3 | “It … it happens with less activity.”  *Interviewer: So, even when you're less active, it still comes on as well?*  “Yeah, a little bit.” |
| 4 | “Yeah, I’m definitely experiencing some [pain]. Like, in my feet when I’m walking. Like, not for a long time, even when I just walk to uni for less than 10 minutes … Yeah, it’s just literally walking like, the first few steps I take out the door.”  “Well, if I walk like, slowly then it’s not so bad, maybe like a 3 or a 4 [over 10] but if I … well, maybe like a 3. If I walk faster, then if I’m like, in a rush then it will be like a 6ish sometimes … it’s just painful like on my foot quite badly when I’m walking.”  “It’s in my feet. Sometimes in my like, legs as well. Like … well, below the knee but mostly in the feet and it’s mostly in the right foot as well.”  “And sometimes my chest as well just gets a bit … like the centre of my chest gets a bit like, I dunno [sic], painful. Like the … in the … not the heart or anything, but like in my ribs, if my … if I’m pushing it against something it just … yeah, hurts quite a bit.” |
| 5 | “I can feel it more, depending on the activity that I’m doing.” |
| 6 | “Well, after not taking medication for some months, I felt some pain in my knees.” |
| **Stiffness** | |
| 1 | “Yeah, so it’s just kinda gone in like a direct positive correlation really. It’s … everything’s … like the pain, stiffness, it’s all increased a lot and yeah, it’s just … it’s a lot more prominent in my hips now and being stiff is, and my ankles as well, whereas it was, it was there before, but it wasn’t to the level it was now and like it eventually it would wear off throughout the day.”  “The stiffness just … previously it was just in the morning whereas now it’s kind of into late afternoon before it frees up.” |
| 2 | *Interviewer: Okay. That’s fine then. Alright. I think you also mentioned that you did not have any stiffness. Can I just ask if there’s been any change in that? Do you, do you have stiffness now or is it still no stiffness?*  “No.” |
| 3 | “… but it’s stiffness, instead of pain.”  *Caregiver: So, it's [stiffness] the same kind of thing, it comes on quicker … Or you get it more intensely?* “… Yeah.” *Caregiver: And more days in the week as well?* “Yeah.” |
| 4 | “I think my left knee is a little bit stiffer than my right knee and I have been kind of noticing that a little bit in the gym . It feels like I can’t move my left leg quite as much as my right sometimes but it’s not like an extreme amount, but it does definitely get annoying with some things and gets in the way.” |
| 5 | “… I get quite stiff in my hip and my knees as well and sometimes, it can affect my drama work.”  *Interviewer: Affects what, sorry?*  “Drama, because I move around a lot.”  …  *Interviewer: So as far as you’re concerned, your stiffness hasn’t changed at all from before?*  “No.” |
| 6 | *Interviewer: Right. What about the other symptoms such as stiffness, fatigue or daytime sleepiness, were they somehow impacted?*  “No.” |
| **Fatigue** | |
| 1 | “Yeah, I think so fatigue as in like mental fatigue definitely has increased and physical fatigue as well, has increased.”  “My main stiffness and pain’s gone up but the, the fatigue and tiredness hasn’t overly changed.” |
| 2 | *Interviewer: … about the tiredness and fatigue, so when you described it the last time, you spoke about it in terms of when you walked for a, a while, you feel that tiredness or fatigue and then you tend to walk a bit slower and then you spoke about the ache. From what you’re saying, that still seems to be happening, but would you think it’s more of a pain and not a tiredness or fatigue?*  “Well, the pain isn’t there every day. So, I think it’s like just being quite tired.” |
| 3 | *Interviewer: And is it similar to what happens with the pain and stiffness where, you know, you don't have to do as much and you still … and you're starting to feel tired and fatigued?*  “Yeah.”  *Interviewer: has the tiredness and fatigue actually prevented you from doing anything, that you want to do, at the minute?*  “Not really, no.” |
| 4 | *Interviewer: Okay. Now, what about the tiredness and fatigue? So, you previously mentioned that yeah, you did feel a bit more tired compared to your friends and again, it was activity related, how has that been now?*  “I think it’s a little bit worse …” |
| 5 | *Interviewer: So you did not have any, or did not report any in the last interview. Has that changed at all?*  “Um, tiredness?”  *Interviewer: Mm hmm.*  “No, it’s not changed (*laughter*).” |
| 6 | *Interviewer: Right. What about the other symptoms such as stiffness, fatigue or daytime sleepiness, were they somehow impacted?*  “No.” |
| **Physical activity** | |
| 1 | “I used to play three, three or four times a week, whereas now I only play once. Just because I, I can’t move [laughs].”  “To give you an example. So before like I used to be able to run upstairs kind of thing, whereas now, it’s just no, I’m not even gonna [sic] try it, ‘cause [sic] I’ll probably end up falling down … ‘cause [sic] … yeah, and then my feet, yeah, ‘cause [sic] the stiffness is there a lot more so yeah, I just can’t move as fast in my legs, yeah.” |
| 2 | “I think I am walking more than I am used to. My parents don’t give me a lift to … from school to home as much as … Because, because my parents are encouraging me to walk more. It’s just that like I get encouraged … to walk more. I have like, like friends that go the same way as me, so I have them to walk with.” |
| 3 | *Interviewer: Do you find that with the pain and the stiffness now being worse, that it's affected the things that you can do?*  “Not really.”  *Interviewer: I know, you're saying you don't really have that much to do now, but has the tiredness and fatigue actually prevented you from doing anything, that you want to do, at the minute?*  “Not really, no.” |
| 4 | “I do still like going, like walking, you know, travelling to … well, yeah, walking to places. I don’t … it’s not like I don’t wanna (sic) walk anywhere anymore. I still do … Yeah, I do enjoy walking, so it’s not really changed that. And the gym, it’s not really affected that either.” |
| 5 | *Interviewer: So it does affect that movement that you need to do?*  “Yes … Usually just drama, because I’m moving around, like all over.”  *Interviewer: And so aside from interfering with your drama club, is there anything else that you find difficult or you can’t do because you’ve now got this pain?*  “Um, no, not really; apart from like maybe bending down, but that’s it.”  *Interviewer: OK, so it doesn’t affect like walking or you know, your daily activities?*  “No.” |
| 6 | No information |
| **Emotional state** | |
| 1 | “But it’s, yeah, I feel very … It’s very frustrating because the effect the injections had on my life is immeasurable, it’s been so … The, the difference now is insane between being on the injection and not being on the injection … That’s probably one of the most frustrating things in terms of not being able to have that injection to make the pain go, go away and prevent it from happening, ’cause [sic] yeah, obviously you get used to certain things and then them things are taken away and pain intensifies. It’s not nice … it’s more frustration at the fact I can’t have the, the injection, yeah. It’s not, yeah, it sounds a bit … Really, it's not frustration in like in my … in my condition, or my legs. But it’s just more of a frustration now that there is something out there that has helped me a lot and will continue to help other people like that feel the same, but unfortunately, it’s just not licenced for adults, so yeah, it’s not really a frustration at myself anymore.”  “So, like aside from being in pain obviously, it’s quite frustrating and ‘cause [sic] when, you know, when you need to, to like run, run for a train or something like that, you can’t, it’s like oh great. But yeah, it’s just frustrating really when you can’t move as you’d like to move.” |
| 2 | “I kind of was happy about being off injections … Because I didn’t … I don’t really like injections that much.” |
| 3 | *Interviewer: you've said pain and stiffness is also worse, has that affected you emotionally in any way, do you feel more upset or frustrated or annoyed, things like that?*  “I don’t think so.” |
| 4 | Not mentioned in the interview |
| 5 | “I feel like obviously, my life would be better off with it [burosumab], but I feel like I’ll be able to live with it, because I have been doing and I don’t think it’s … I don’t think it’s going to get any worse than this … I feel like I can be content with not being back on it, because I can live with it, but I would rather be back on it.”  *Interviewer: So how do you feel about it? Because you didn’t have pain the last time, so how do you feel about that?*  “Annoyed. Yes, I don’t like the fact that I can’t do things that I used to do any more.  Sometimes, when I’m stiff or when I’m in pain, I feel like I look like I’m walking a bit weirdly, because obviously, I don’t want it to hurt as bad and I feel quite self-conscious when I’m doing that, because I feel like it stands out.”  *Interviewer: Mm hmm. And so does that affect both your self-confidence and your self-esteem?*  “I feel like just my confidence.”  *Interviewer: It’s a little bit lower than …*  “Yes.” |
| 6 | *Interviewer: Thinking about the change that you have just described regarding pain; how does that make you feel? How did that make you feel?*  “Um …”  *Interviewer: For instance: sad, anxious, angry …*  “Um … no. I felt the same.” |

Supplementary Figure 1 Individual symptom figures and daily step count for adolescents who discontinued burosumab treatment after EoSG


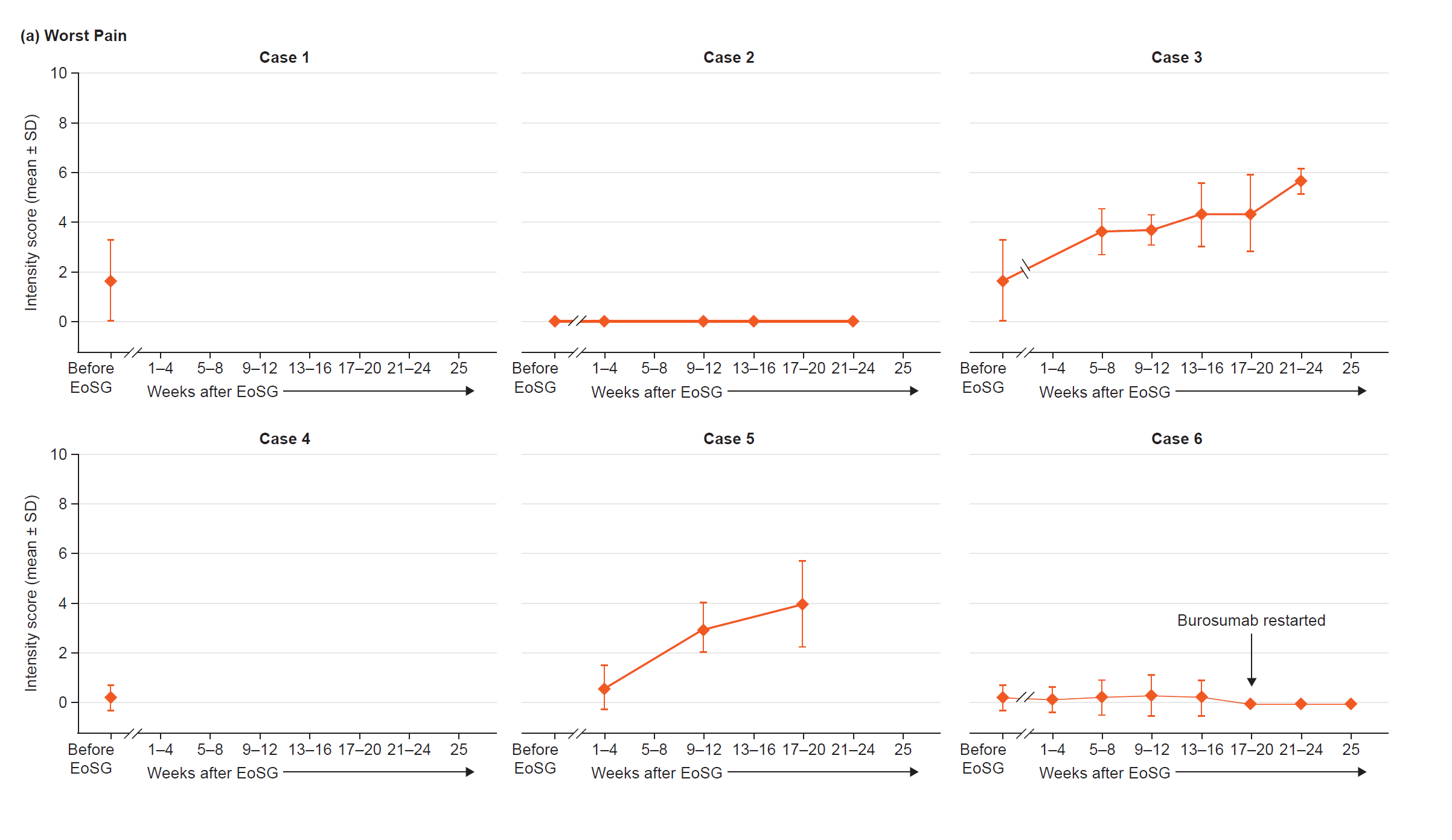


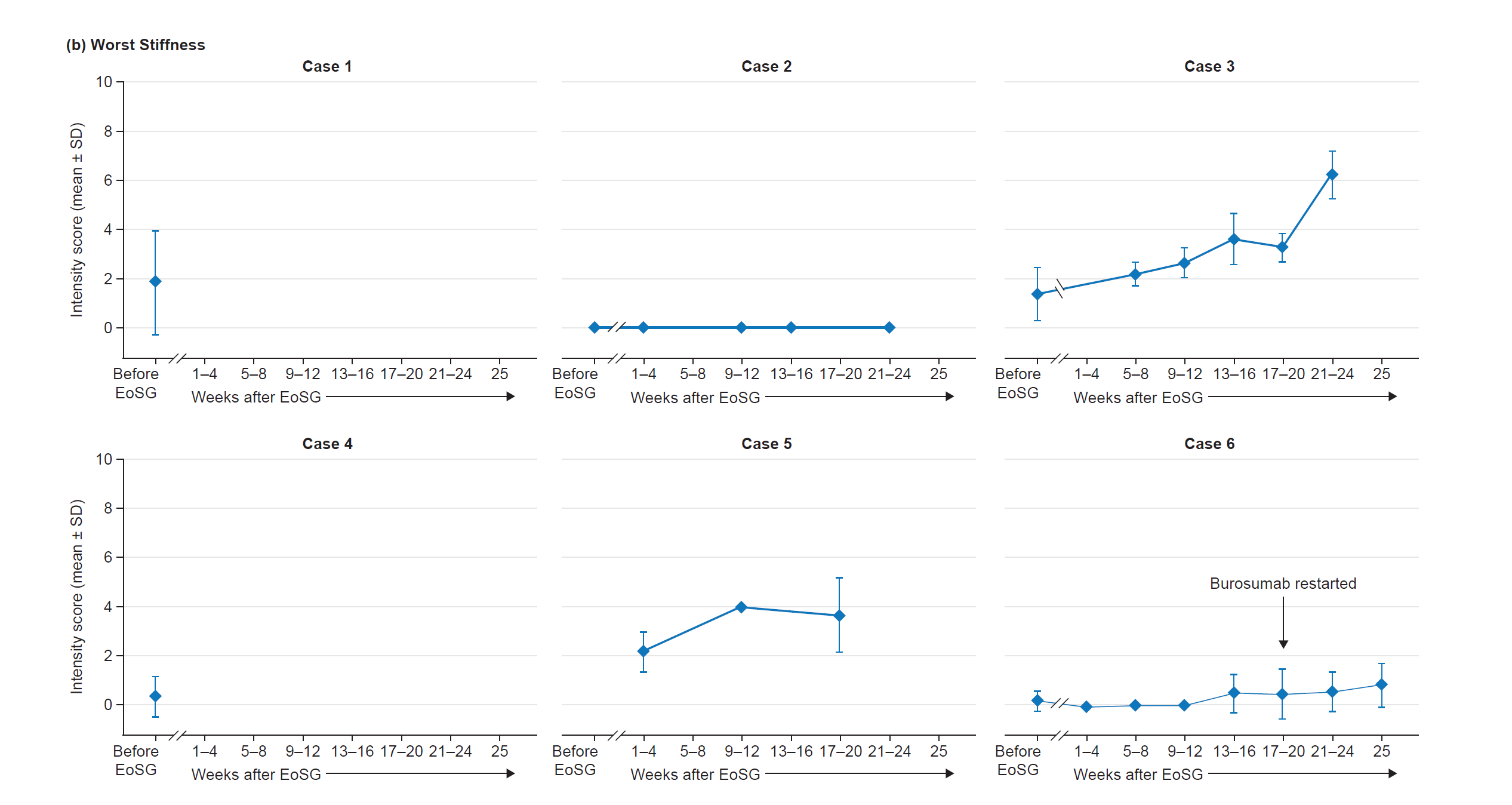


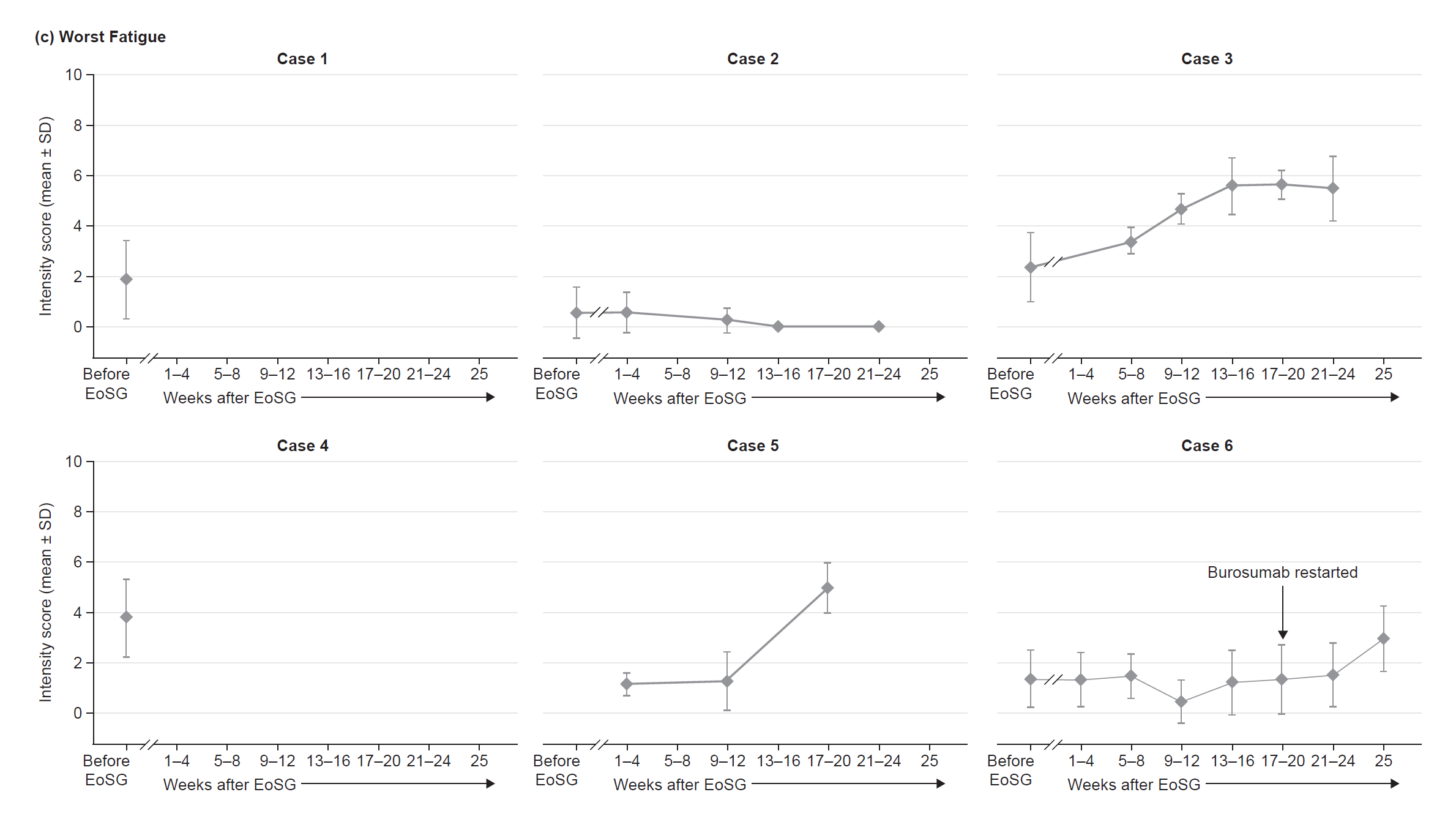


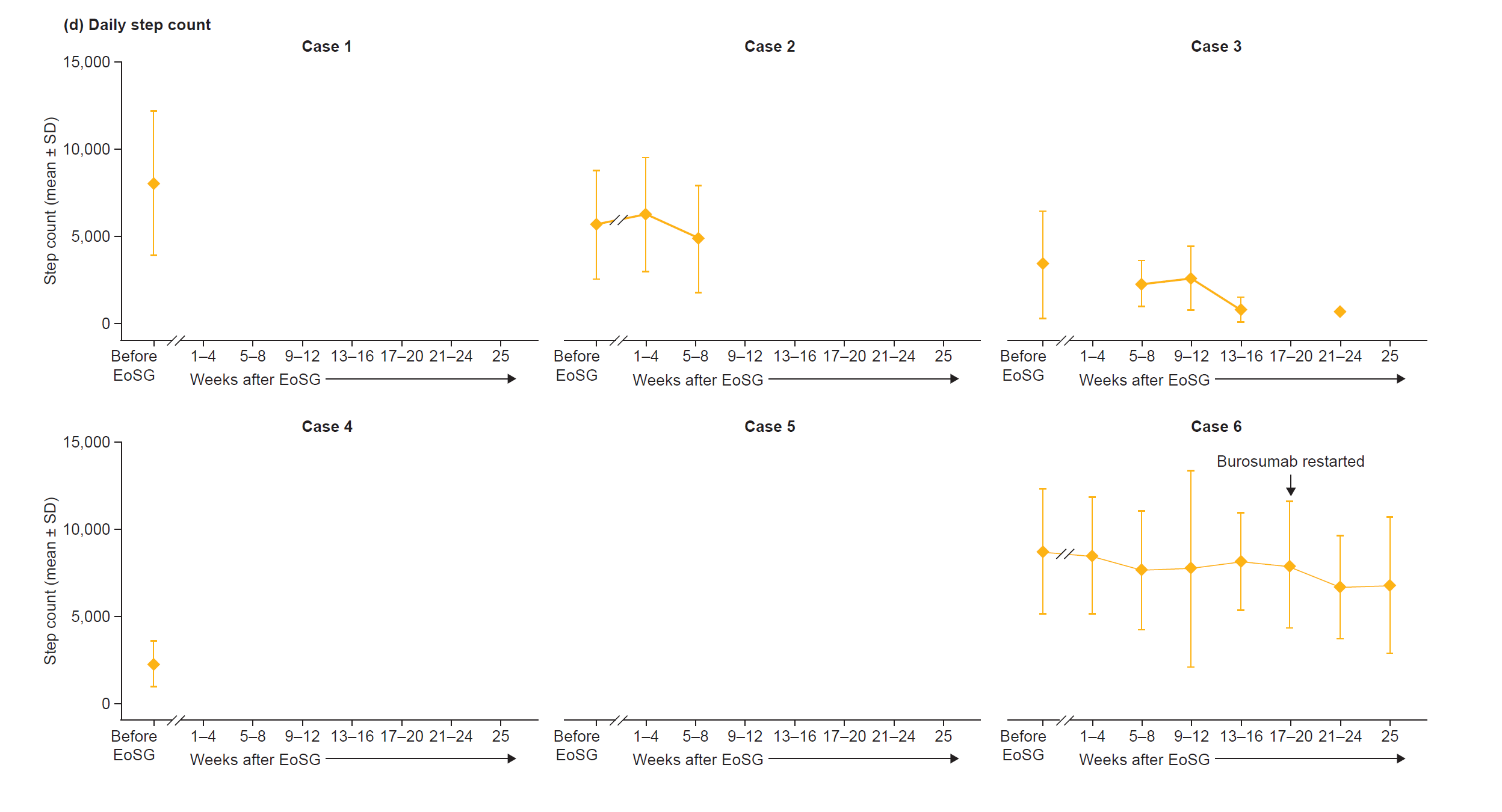


EoSG, end of skeletal growth; SD, standard deviation
